# Age-dependent electrical and morphological remodeling of the *Drosophila* heart caused by hERG/*seizure* mutations

**DOI:** 10.1371/journal.pgen.1006786

**Published:** 2017-05-19

**Authors:** Karen Ocorr, Alexander Zambon, Yoav Nudell, Santiago Pineda, Soda Diop, Min Tang, Takeshi Akasaka, Erika Taylor

**Affiliations:** Development, Aging and Regeneration Program, Sanford-Burnham-Prebys Medical Discovery Institute, La Jolla, California, United States of America; Stanford University School of Medicine, UNITED STATES

## Abstract

Understanding the cellular-molecular substrates of heart disease is key to the development of cardiac specific therapies and to the prevention of off-target effects by non-cardiac targeted drugs. One of the primary targets for therapeutic intervention has been the human ether a go-go (hERG) K^+^ channel that, together with the KCNQ channel, controls the rate and efficiency of repolarization in human myocardial cells. Neither of these channels plays a major role in adult mouse heart function; however, we show here that the hERG homolog *seizure* (*sei*), along with *KCNQ*, both contribute significantly to adult heart function as they do in humans. In *Drosophila*, mutations in or cardiac knockdown of *sei* channels cause arrhythmias that become progressively more severe with age. Intracellular recordings of semi-intact heart preparations revealed that these perturbations also cause electrical remodeling that is reminiscent of the early afterdepolarizations seen in human myocardial cells defective in these channels. In contrast to *KCNQ*, however, mutations in *sei* also cause extensive structural remodeling of the myofibrillar organization, which suggests that hERG channel function has a novel link to sarcomeric and myofibrillar integrity. We conclude that deficiency of ion channels with similar electrical functions in cardiomyocytes can lead to different types or extents of electrical and/or structural remodeling impacting cardiac output.

## Author summary

We have used the fruit fly cardiac model to show that *seizure*, the fly homolog of the human ether a go-go K^+^ channel hERG, is functional in the fly heart. This channel plays a major role in cardiac repolarization in humans but not in adult rodent hearts. Loss of channel function in the fly causes bradycardia, electrical arrhythmia and altered myofibrillar structure. Gene expression analysis indicates that Wnt signaling is affected and we show a genetic interaction between *sei* and *pygopus*, a Wnt pathway component, on heart function.

## Introduction

Cardiac performance significantly impacts quality of life and heart disease accounts for more deaths (Centers for Disease Control and Prevention, American Heart Association, World Health Organization) than any other disease. However, much remains to be learned concerning the cellular and molecular mechanisms involved in pathological cardiac remodeling, in order to design newer, more effective treatments. The vertebrate heart is a complex multilayer structure whose autonomous function can be modulated by neurohormonal factors. In addition, genetic, metabolic and environmental factors all contribute to heart disease, thus complicating elucidation of the underlying cellular mechanisms. Complex genetic studies in vertebrate models are difficult to conduct and are not easily interpretable, and studies of the effects of aging, even in mouse models, take years. Significantly, studies in mice are also complicated by the fact that important K^+^ channels that regulate repolarization in the human heart do not make major contributions to heart function in murine adults (reviewed in [1,2]). These channels include the hERG K^+^ channel that is a target for many anti-arrhythmia drugs, as well as the KCNQ K^+^ channel, and mutations in both of these genes contribute to long QT syndrome (LQTS) which can trigger cardiac arrhythmias and are likely significant contributors to sudden cardiac death.

The *Drosophila* heart model has been successfully used to elucidate the molecular-genetic basis of cardiac development and pioneered our understanding of the origins and specification of cardiogenesis in the animal kingdom [3–5]. More recently the fly heart has also become a prototypical model to study the genetic causes of cardiac dysfunction and aging (reviewed in [6–8]). The heart of Drosophila is a linear tube composed of a single layer of myocardial cells [9], which forms in a homologous fashion to the early embryonic heart in vertebrates [10], but remains much less complex than the looped and chambered multilayered adult vertebrate heart. Despite the structural differences, a number of studies indicate that there are significant functional similarities between the fly and human heart. As in vertebrates, heart function in Drosophila is myogenic [11,12] and its rate can be modulated by neuronal and hormonal input [13,14]. Heart muscle protein composition, as well as muscle function and dysfunction share many similarities to human hearts [15–18]. Both dilated and restricted cardiomyopathies have been described by us and others in the fly heart and have been linked to mutations in homologous genes with similar effects in human heart [17,19–22]. In addition, there is evidence of channelopathies in the fly heart that are reminiscent of what is observed in human patients [6,14,16,23–25]. For example, we have previously shown that the KCNQ K^+^ channel, which underlies a slow outward rectifying current in human myocardial cells (I_Ks_), also functions in the fly heart and that mutations in this channel contribute to heart arrhythmias [16], as they do humans ([26–28]).

Here, we show that the hERG homolog *seizure* (*sei*), which is responsible for a rapid repolarizing current (I_Kr_) in human heart, also functions similarly in the fly heart. Mutations in *sei* and adult cardiac-specific knockdown of this channel cause early after-depolarizations and cardiac arrhythmia. In addition, *sei* mutant hearts show considerable morphological remodeling that is not observed in hearts from flies with mutations in the KCNQ voltage-dependent K^+^ channel [16]. Expression analysis suggests that Wnt signaling is misregulated in hearts from *sei* mutants, and that misregulation of this pathway enhances the compromised *sei* function in generating cardiac pathologies. Our results suggest that alterations in *sei* channel function may play novel roles in cardiac remodeling that involves *Wnt* signaling.

## Results

### *sei* expression in the *Drosophila* heart

**Fig 1:**
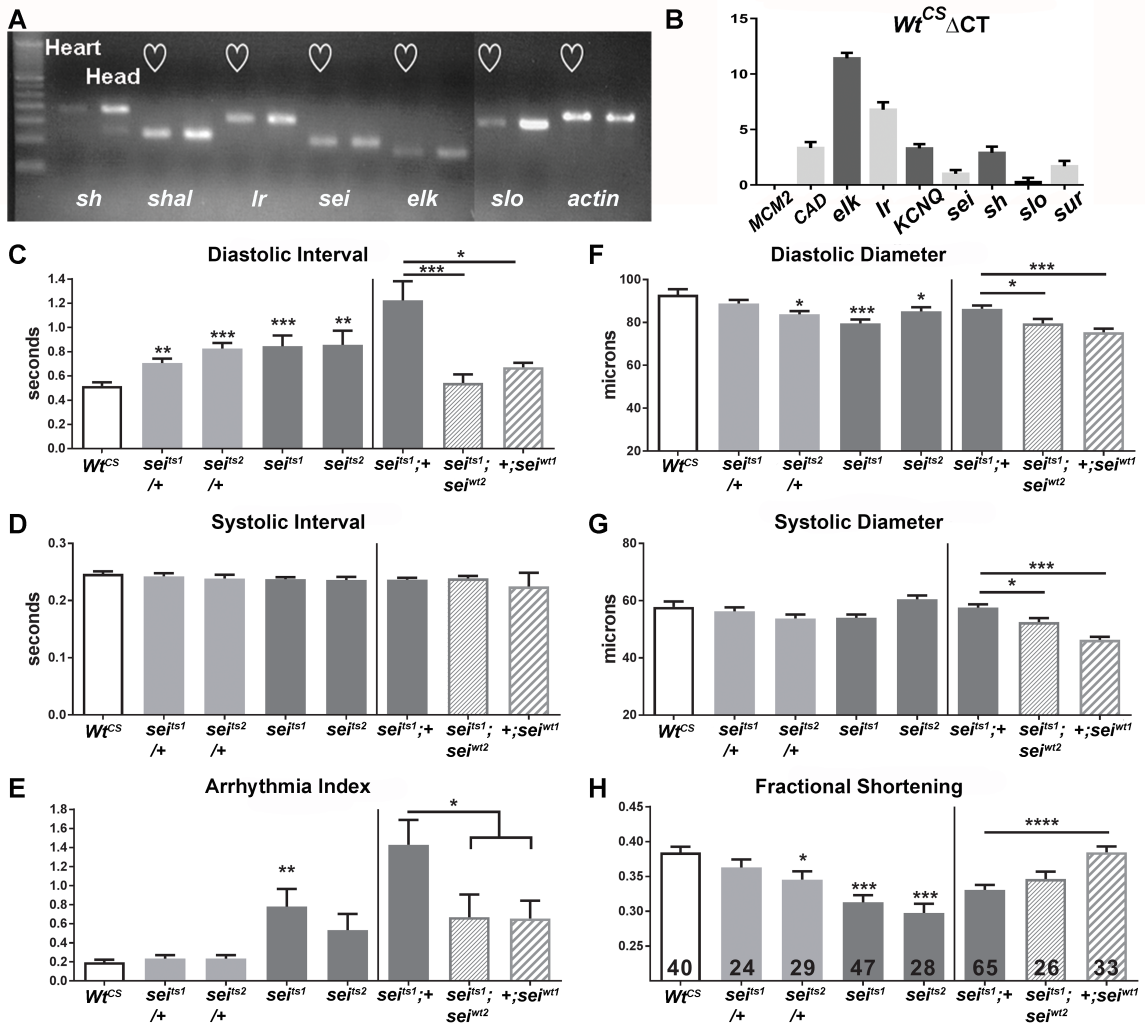
**(A) qPCR of isolated hearts and heads from wildtype (*w1118*) *Drosophila*.** Seven potassium channels, KCNQ, seizure (sei), the BK channel slowpoke (slo), shaker (sh), the inward rectifier (Ir), ether a go-go like (elk) and Shaker like (shal) exhibited significant expression in isolated hearts. Expression in the head (mostly nervous tissue) is shown for comparison. **(B)** Δ Ct values of different voltage-activated K^+^ channels calculated from raw Ct values in the wild-type Canton-S (Wt^CS^) background. Note that ΔCt values are inversely correlated with relative expression such that a channel like *elk* is weakly expressed while the Ca^2+^ channel, *Ca-alpha1D (CAD)*, as well as K^+^ channels *KCNQ* and *sei* have relatively high levels of expression. **(C-E) Functional analysis of *sei* mutants shows alterations in heart function**. (C, left) Mean Diastolic Intervals (DI) were significantly increased in hearts from 1 week old *sei* heterozygotes and homozygote mutants compared to their genetic background control *Wt*^*CS*^. (C, right) The increase in mean DI observed for *sei*^*ts1*^ mutants was rescued by insertion of two genomic copies of wildtype *sei* (*sei*^*wt2*^). Overexpression of an extra copy of wt *sei* in the wt background (+;*sei*^*wt1*^) did not affect DI. (D, left) Mean Systolic Intervals (SI) did not vary among the different genotypes. (D, right) Mean SI did not vary among the genotypes. (E, left) The incidence of arrhythmia (quantified as the heart period standard deviation normalized to the median heart period) was significantly increased in hearts from homozygous *sei*^*ts1*^ homozygous mutants. (E, right) The increase in arrhythmia was partially rescued by insertion of two copies of the wt sei gene (*sei*^*wt2*^). **(F-H) Contractility is impaired in *sei* mutants**. (F, left) Heart diameters during diastole were significantly smaller in one heterozygote and in both homozygous *sei* mutants compared to their background control, Wt^CS^. (F, right) Heart diameters during diastole were significantly smaller in hearts from *sei* rescue and overexpression flies compared to *sei*^*ts1*^ mutant. (G, left) The diameters of the hearts during systole did not vary significantly between the *sei* mutant lines and their background control, Wt^CS^. (G, right) Heart diameters during systole were significantly smaller in hearts from *sei* rescue and overexpression flies compared to *sei*^*ts1*^ mutant. (H, left) Fractional shortening is reduced significantly in both of the homozygous *sei* mutant lines (H, right) but was not rescued in hearts from flies expressing genomic *sei*^*wt*^. For all figures significance was calculated using one-way ANOVA and Dunnett’s multiple comparison post-hoc test.; *p<0.05, **p<0.01, ***p<0.001, ****p<0.0001. Numbers of hearts examined are shown in bars in G.

In human hearts, a number of different K^+^ channels contribute to the repolarization of cardiac action potentials (APs). We used PCR to examine expression of different K^+^ channels in one week old *Drosophila* adults. Hearts and heads were isolated and real time PCR (rtPCR) analysis showed significant expression of a number of K^+^ channels (Fig 1A). These included the voltage-dependent K^+^ channels *Shaker* (*Sh*) and *Shaker-like* (*Shal*). Also fly homologs for the human Ether-a-gogo Related Gene (hERG) were identified, including *seizure* (*sei*) and *ether-a-gogo-like* (*elk*). The Ca^2+^activated K^+^ channel, encoded by *slowpoke* (*slo*), which is homologous to the big K^+^ (BK) channel, was also expressed in the heart. Expression observed for all these channels was at levels somewhat lower to those found in neural tissue (‘head’ in Fig 1A). To confirm this result we used Fluidigm’s Biomark nanofludic qPCR to also measure gene expression levels in isolated hearts (Fig 1B). We used expression of the L-type Ca^2+^ channel, *Ca-alpha1D (CAD)*, which has been shown to underlie the cardiac action potential (AP) in flies,[29] as a positive control. Results show that *KCNQ*, *sei*, *sh*, and the sulfonylurea receptor channel (*sur*, previously shown to play a role in fly heart function) are expressed at roughly the same levels as is *CAD*, with lower expression of *elk* and *Ir*. Thus, including the previously documented KCNQ channel [16] and K_ATP_ channel dSUR [25] the fly heart expresses at least eight different potassium channels all of which are likely to be involved in regulating cardiac repolarization, as they do in mammals and humans[1,30].

### Loss of *seizure* function causes arrhythmias and reduced contractility

Analysis of high speed movies of beating hearts combined with our Semi-automated Heartbeat Analysis (SOHA) software (www.sohasoftware.com) allows precise quantification of a number of cardiac function parameters[31,32]. Heart function in the fly, as in humans, is myogenic and modulated by neuronal and hormonal input (Dlucis & Levine 1997). Hearts from young (1 week old) wildtype (wt) flies, when denervated as in the semi-intact preparations [16], typically beat with a regular endogenous rhythm of 1–3 Hz; this preparation allows us to monitor effects on myocardial cells in the absence of CNS input. In the absence of innervation the heart contraction/relaxation events (systolic intervals, SI) in young flies are between 0.2–0.28 seconds and relaxation periods (diastolic intervals, DI) are between 0.5–0.8 seconds (e.g. [16,18]) with DIs tending to be much more variable than SIs.

Our analysis of heart function in both *sei* mutants, *sei*^ts1^ and *sei*^ts2^, revealed that mutant hearts exhibit a significantly slowed heart rate, compared to that of the Canton S genetic background controls (*Wt*^*CS*^). This bradycardia was due primarily to a lengthening of the DI that is apparent, even in heterozygotes (*sei*^*ts1*^;+ and *sei*^*ts2*^;+, Fig 1C-left), with little change in the SI length (Fig 1D-left). *sei* mutant flies also exhibited an increase in arrhythmic beating compared to controls, whereas this parameter was unaffected in heterozygotes (Fig 1E-left). In addition, we observed a significant reduction in diastolic diameters (Fig 1F-left), but not in systolic diameters (Fig 1G-left), when compared to hearts from controls. We also observed significant decreases in cardiac contractility, measured as fractional shortening (FS), in *sei* homozygous mutants whereas hearts from heterozygotes showed only mild or no reductions in fractional shortening (Fig 1H-left). Similar alterations in heart function parameters were also seen in hearts from 3 and 5 week old homozygous mutant flies (S1 Fig).

In order to unequivocally demonstrate that the heart defects observed in mutants are due to a dysfunctional *sei* locus, we attempted to rescue the cardiac abnormalities of the *sei* mutation with wt genomic copies of this locus (see Materials and methods) in a *sei*^*ts1*^ mutant background. Hearts from homozygous *sei*^*ts1*^ flies crossed into the rescue construct background (*sei*^*ts1*^;+) showed the expected bradycardia due to increased DI. This bradycardia was rescued by the introduction of two genomic copies of the wt gene (*sei*^*ts1*^;*sei*^*wt2*^, Fig 1B-right). Similarly the increased arrhythmicity observed in response to mutation of the *sei* gene could be partially rescued by the introduction of two wt *sei* genes (Fig 1D-right). However, the reductions in heart diameters and contractility (FS) that we observed in hearts with two copies of the mutant gene were not rescued by the addition of genomic copies of *sei* (Fig 1E–1G, right); this may be due to the particular insertion site of the rescue construct causing suboptimal expression. An extra genomic copy of the sei gene in the wt rescue background (+,*sei*^*Wt1*^) by itself had little effect on heart function compared to controls (Fig 1C–1H).

**Fig 2:**
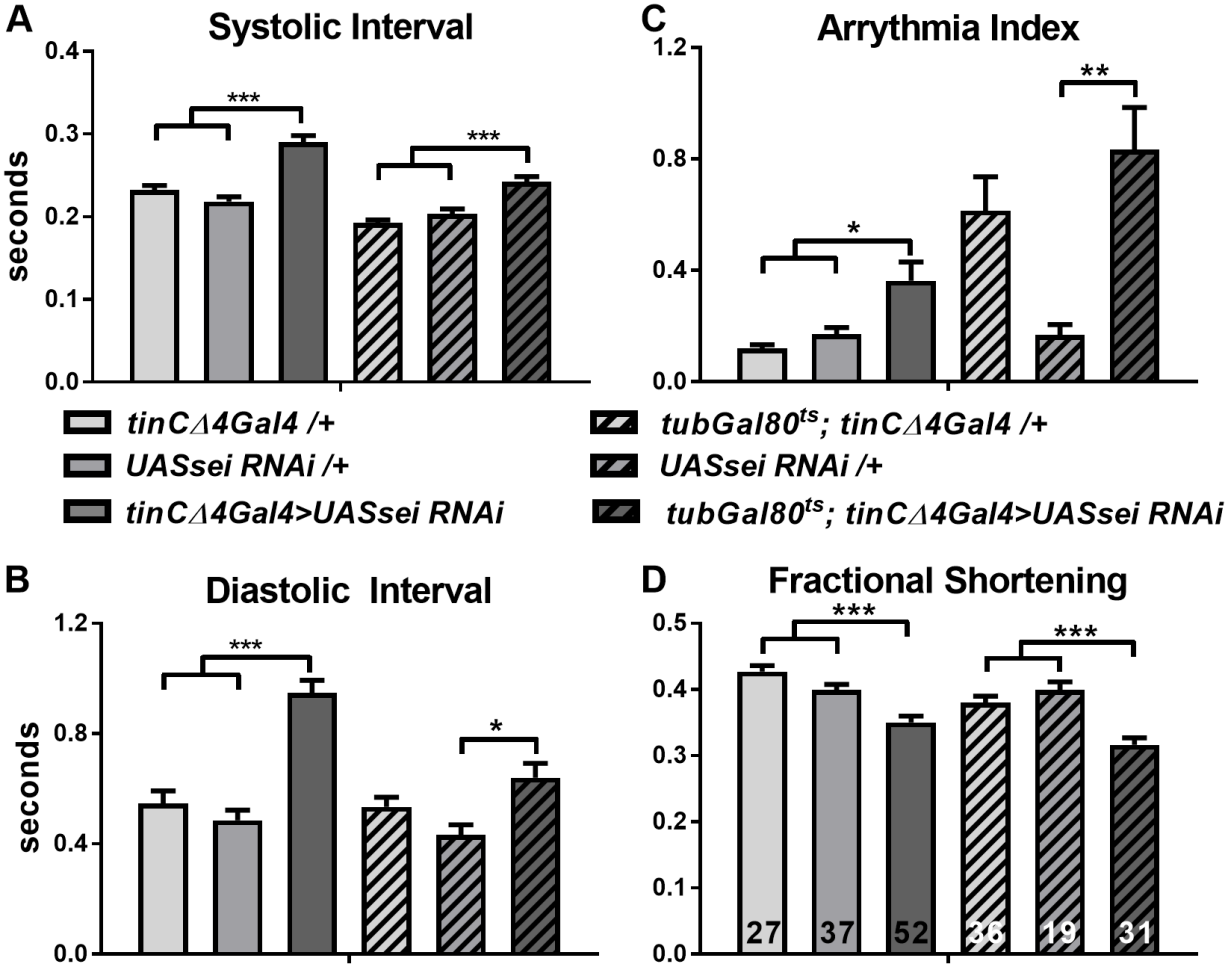
Heart and adult-specific knockdown of *sei* affects heart rhythmicity and contractility. Heart-specific KD of *sei* was achieved by crossing flies with a UAS*sei* RNAi construct to the heart-specific driver tinCΔ4 Gal4. Adult specific KD was achieved using a temperature-sensitive driver tub-gal80^ts^ in combination with tinCΔ4 Gal4 to knock down *sei* specifically in the adult heart (see Materials and methods). **(A&B)** Heart-specific (tin and adult specific KD of *sei* causes a significant increases in both systolic (A) and Diastolic Intervals (B) compared to controls (tinCΔ4 driver/+, tub gal80^ts^; tinCΔ4 driver/+, and UAS*sei* RNAi /+) at 3 weeks of age. **(C)** Heart-specific and adult specific KD of *sei* causes a significant increases in arrhythmia compared to controls (tinCΔ4 driver/+, tub gal80^ts^; tinCΔ4 driver/+, and UAS*sei* RNAi /+) at 3 weeks. **(D)** The fractional shortening, an indicator of heart contractility, was significantly reduced in 3 week *sei* KD hearts compared to controls. Significance was calculated using a two-way ANOVA and Tukey’s post-hoc test; *p<0.05, **P<0.01,****p<0.0001; number of flies examined is shown in bars in D.

Because the *sei* channel plays a role in nervous system function we wanted to confirm a heart autonomous role for this ion channel. We crossed a cardiac-specific driver (*tinCΔ4-Gal4*) with UAS-*sei-*RNAi flies to specifically knockdown (KD) *sei* in myocardial cells. We examined heart-specific *sei* KD at three weeks of age because our structural data suggested that the effects of *sei* mutations were age-dependent (see below); thus the cardiac phenotype would be expected to be more robust in KD hearts compared to wt hearts which do not exhibit many functional defects at 3 weeks. Cardiac *sei* KD caused increases in the DI as was seen in *sei* mutants and also caused significant increases in the SI (Fig 2A & 2B, solid bars). In addition, there was a significant increase in arrhythmia (Fig 2C, solid bars). Hearts also showed a significant decrease in fractional shortening by three weeks of age (Fig 2D, solid bars), as for both systemic *sei* mutants (S1 Fig).

We also used a heart-specific temperature-sensitive Gal4 driver (*tubGal80*^*ts*^; *tinCΔ4-Gal4*) to determine whether KD only during adult stages was sufficient to generate the observed heart defects, or whether developmental effects also played a role. Flies were cultured at the permissive temperature of 18°C until eclosion, at which time the adult flies were collected and switched to 25°C. At this non-permissive temperature Gal80 is no longer expressed and its inhibitory effects on Gal4 activity are removed. Hearts from 3 week old adults, maintained at 25°C, showed significantly increased SI, DI and AI, compared to driver and *UASsei RNAi* only controls exposed to the same temperature regime (Fig 2A–2C; striped bars) and we again observed a significant decrease in fractional shortening (Fig 2D; striped bars).

In sum, we found that that *sei* plays a role in heart function in the fly and compromised *sei* function in the adult heart causes bradycardia even at young ages. In contrast to *KCNQ* mutants, we find that impaired *sei* function causes reduced contractility and causes age-dependent morphological remodeling, suggesting that *sei* may have broader consequences than just affecting electrical properties of the heart.

### *Seizure* and *KCNQ* mutations have differential effects on cardiac contractility under loaded conditions

**Fig 3:**
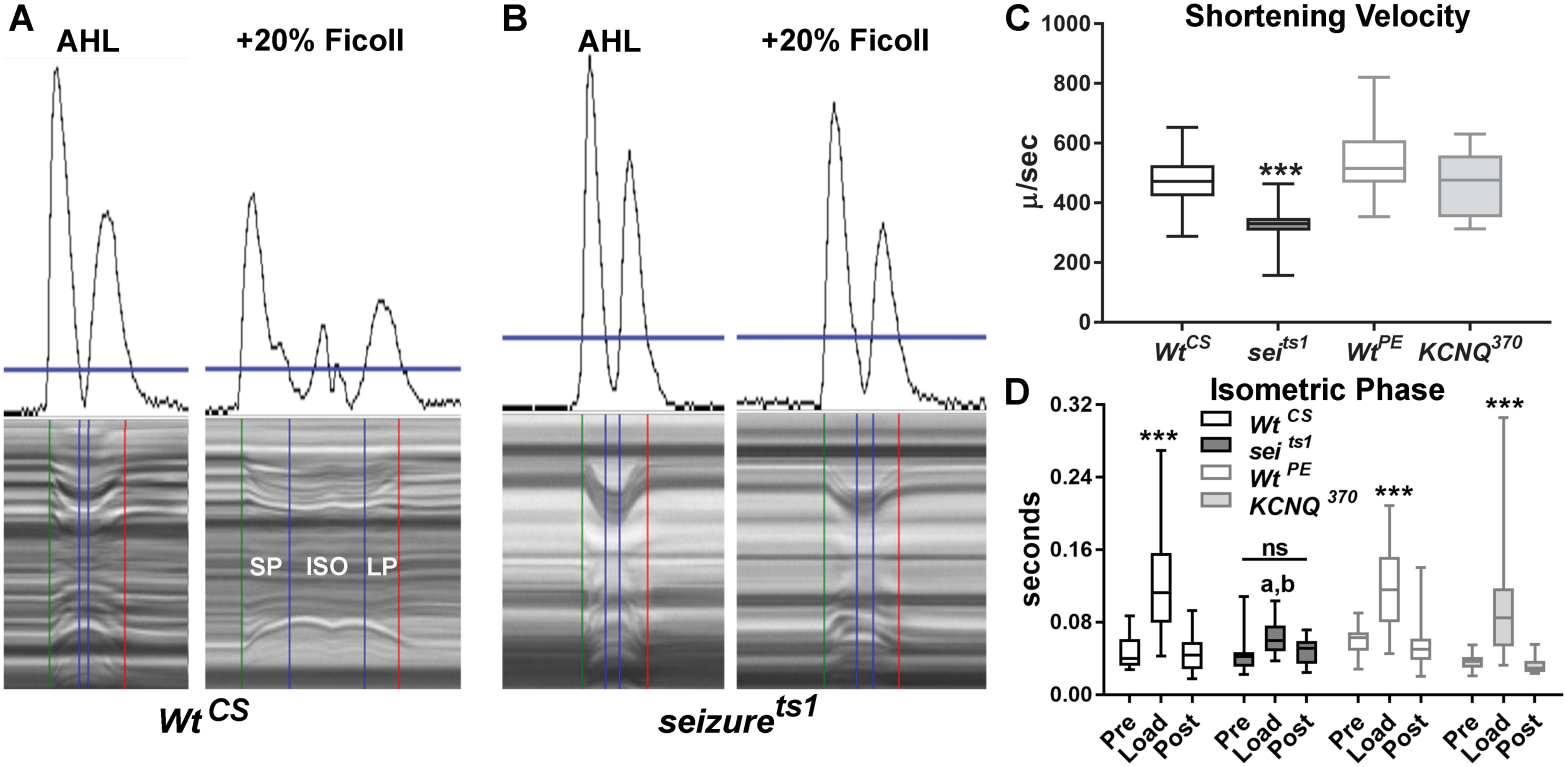
Cardiac contractility under loaded conditions. **(A-AHL)** Movement traces (top) and corresponding M-mode (bottom from a *Wt*^*CS*^ heart under unloaded conditions (normal AHL). Note that there are distinct movement peaks produced by the contraction and relaxation of the heart wall that are used along with a movable threshold bar (horizontal blue line in A & B) to accurately parse a single contraction into shortening (SP), isometric (ISO) and lengthening phases (LP). **(A-+20% Ficoll)** Hearts beating for 30 min under load (AHL + 20% Ficoll 400) show a prolonged shortening phase (SP) and an obvious isometric contraction phase (ISO). **(B)** Hearts from *sei*^*ts1*^ mutants do not exhibit a prolonged ISO phase under load. **(C)** Shortening velocities were calculated as (Diastolic Diameter-Systolic diameter)/ Shortening Phase, SP) see Cammarato et al, 2015. Under unloaded conditions *Sei*^*ts1*^ mutants showed significantly reduced shortening velocities compared to *Wt*^*CS*^ as well as to *Wt*^*PE*^ and *KCNQ*^*370*^ (a K^+^ channel mutant). **(D)** The length of time that hearts maintained isometric contractions was significantly increased under Load (plus 20% ficoll) for all genotypes except for *sei* mutants (ns = not significant; a-p<0.0001 with Wt^CS^ and Wt^PE^; b-p<0.05 with KCNQ^370^). For C&D data are shown as the mean, 2.5–97.5% confidence interval, Maximum and Minimum values. Significance in (C) was determined using a one way ANOVA and in (D) using a two-way ANOVA, both with Tukey’s multiple comparisons post-hoc test, **p<0.01, ***p<0.001. (*Wt*^*CS*^ n = 16, *sei*^*ts1*^ n = 23, *Wt*^*PE*^ n = 24, *KCNQ*^*370*^ n = 16).

To explore the roles of *sei* and *KCNQ* in the heart further, we used a method we have recently developed for monitoring contractility under conditions that put a load on the fly’s heart using artificial hemolymph of increasing viscosity [32]. This methodology allows us to automatically parse individual contractions into three distinct phases: shortening (SP), isometric (ISO) and lengthening phases (LP, Fig 3A). In addition, we can calculate shortening velocities in conjunction with measurements of heart wall movement distances. Assays done under unloaded conditions (normal artificial hemolymph) and then loaded conditions (plus 20% Ficoll) allow us to examine relative force production by the heart.

Under unloaded conditions, mean shortening velocities for *KCNQ* null mutants (*KCNQ*^*370*^, see Ocorr et al, 2007) were not significantly different from that of *Wt*^*PE*^ hearts (the precise excision genetic background control, Fig 3C). However, *sei* mutants showed a significantly reduced shortening velocity compared to *Wt*^*CS*^, the *sei* genetic background control, as well as compared to hearts from *Wt*^*PE*^ and *KCNQ* mutants. This reduced velocity was due to in part to the decreased FS exhibited by the *sei* mutants. The length of time that hearts were able to maintain an isometric contraction under unloaded conditions did not vary between genotypes (Fig 3D–“Pre”). However, under loaded conditions hearts from both control flies and *KCNQ*^*370*^ mutants exhibited significantly increased isomeric contraction times (Fig 3D–“Load”, 20% Ficoll). In contrast, *sei*^*ts1*^ mutants appeared unable to increase isometric contraction times under a load, suggesting a defect in the ability of these hearts to generate tension. Hearts from all four genotypes recovered to pre-load values 30 min after the load was removed (Fig 3D- “Post”). These finding further support the notion that sustained *sei* deficiency affects contractility more than a loss of *KCNQ*.

### Electrophysiological analysis of young *sei* and *KCNQ* mutants show early afterdepolarizations that is similar to electrical activity seen in aged wt flies

To monitor the intrinsic electrical activity of myocytes in the cardiac tube, we used standard intracellular recording techniques with sharp electrodes. In all experiments (except where specifically indicated) we used a pretreatment with 10uM blebbistatin to inhibit muscle contraction [33], which under the conditions used does not significantly affect electrical activity [34]. Although reversible, this pretreatment blocked contractions for extended periods following removal of blebbistatin and permitted us to obtain recordings that were stable and could be maintained in some cases for as long as one hour. Inhibition of active contractions would be expected to minimize damage to the cell membrane at the point of electrode penetration and to prevent movement artifacts. All hearts examined under these conditions exhibited spontaneous electrical activity, as expected of myogenic heart tissue in a denervated preparation.

**Fig 4:**
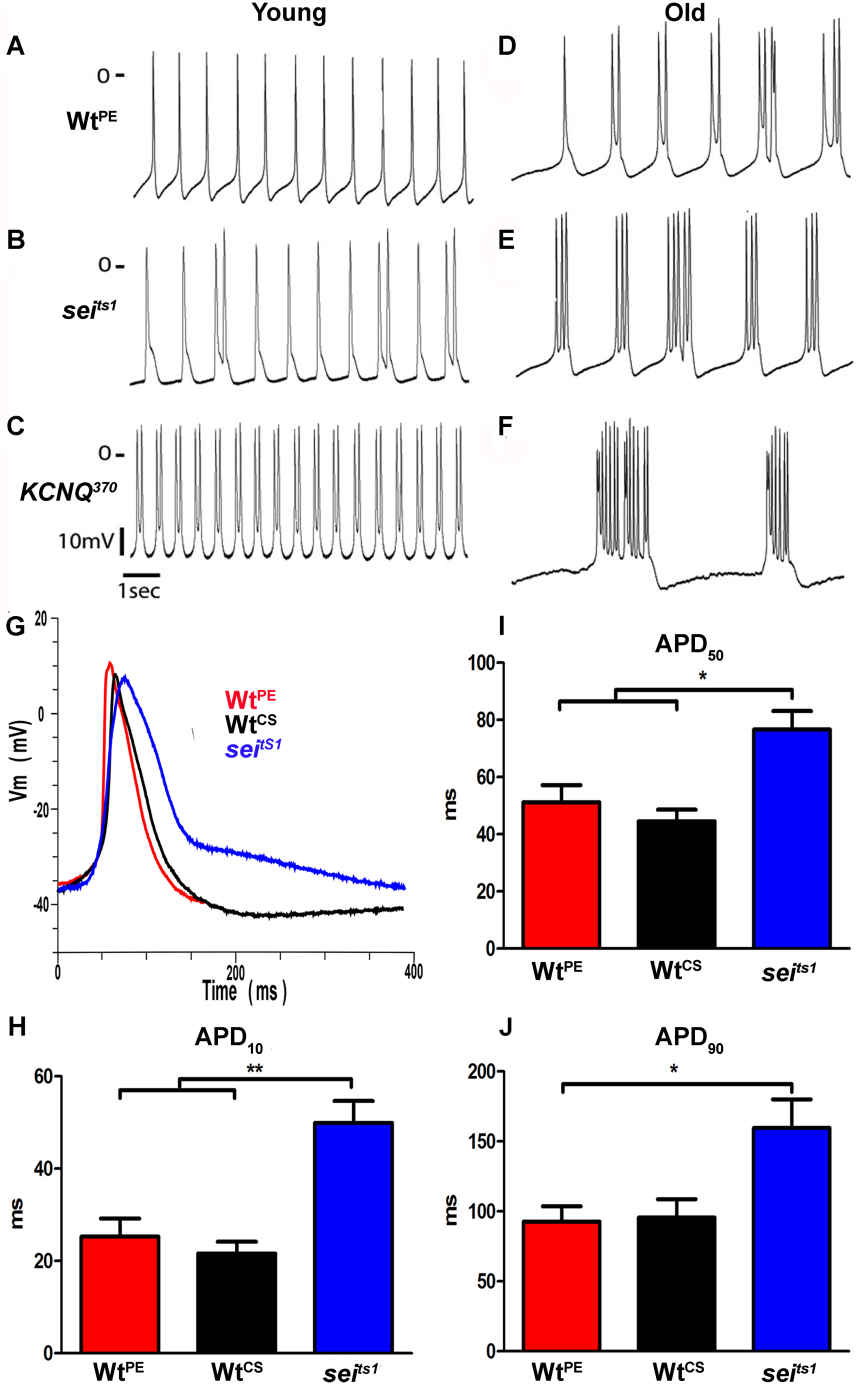
Spontaneous action potentials from *Drosophila* myocardial cells. Action potentials were recorded using intracellular recording techniques from fly hearts in which contractions had been blocked with 10uM blebbistatin. **(A,C,E)** Action potentials from myocardial cells in hearts from young flies (1–2 week old). **(B,D,F)** Action potentials from myocardial cells in hearts from old flies (4–6 weeks). **(G)** Superimposed action potentials from two wt lines (*Wt*^*PE*^-red; *Wt*^*CS*^-black) and *sei*^*ts1*^ mutant (blue) hearts. **(H-J)** Action Potential Duration (APD) was measured for single-peak APs at the point where the membrane voltage had repolarized to 10% (H), 50% (I) and 90% (J) of its maximum depolarized potential. (one-way ANOVA;*p<0.05; ** p<0.01).

Recordings from myocardial cells in hearts of young (1–2 week old) wt flies lines show regular overshooting action potentials (APs) with amplitudes around 50 mV (Fig 4A, Table 1). Resting membrane potentials ranged between -50 and -35 mV; these values in adult hearts are significantly more hyperpolarized than previously reported for intracellular recordings from larval hearts [35,36]. Recordings from myocardial cells in *sei* mutant hearts often exhibited APs with two peaks (Fig 4B, Table 1), likely triggered by early afterdepolarizations (EADs) as has previously been described in vertebrate myocardial cells [37]. Hearts from both *sei* and *KCNQ* mutants typically exhibited bursts of APs but the frequency of this type of event was higher for *KCNQ* compared to *sei* mutants (Fig 4; Table 1 quantified as “Peaks/Burst” and “Event Duration”). Triggered APs were rarely observed in young wt hearts (Table 1, Fig 4A). In addition, single peak APs from *sei* mutant hearts exhibit dramatically broadened APs compared to both wt lines (*Wt*^*PE*^ and *Wt*^*CS*^, Fig 4G) at points where repolarization is 10% (APD_10_) and 50% (APD_50_) complete (Fig 4I and 4H), and significantly broader than *Wt*^*PE*^ APs at 90% repolarization (APD_90_) (Fig 4J, Table 1). We were unable to record single peak APs from *KCNQ* mutants so they were not included the AP duration analysis.

**Table 1:** Intracellular electrical activity.

	N	Resting V_m_ (mV)	Maximum Amplitude (mV)	Peaks / Burst	Event Duration (ms)	APD_10_ (ms)	APD_50_ (ms)	APD_90_ (ms)
**Wt**^**PE**^	**7**	**-42.8 ± 1.6**	**48.4 ± 3.2**	**1**	**136 ± 31**	**25.27± 3.95**	**51.18 ± 6.02**	**92.77 ± 10.92**
**Wt**^**CS**^	**5**	**-39.9 ± 0.1**	**48.8 ± 1.5**	**1.16 ± 0.2**	**106 ± 16**	**26.13± 7.01**	**50.07 ± 9.72**	**92.97 ± 17.74**
***Sei***^***ts1***^	**9**	**-38.5 ± 3.6**	**53.3 ± 2.9**	1.5 ± 0.2*	388 ± 43 ***	48.04 ± 3.88**	74.64 ± 5.31*	**146.3 ± 22.17**^**%**^
***KCNQ***^***370***^	**5**	**-40.4 ± 5.4**	**53.3 ± 6.7**	7.9 ± 2.2**	1338 ± 338 ***	**No Single-Peak APs were observed**		
***tinCΔ4Gal4 / +***	**5**	**-41.7 ± 1.2**	**51.0 ± 2.0**	**1**	**211 ± 17**			
***UASseiRNAi / +***	**6**	**-37.0 ± 1.9**	**47.8 ± 1.5**	**1.5±0.2**	**226 ± 46**			
***tinCΔ4Gal4 >UASseiRNAi***	**5**	**-41.82 ± 5.2**	**58.2 ± 3.6**	**3.8 ± 0.2**^**#**^	**616 ± 44**^**##**^	**No Single-Peak APs were observed**		

Hearts from 1–2 week old flies were analyzed using standard sharp electrode intracellular recording techniques. Data is presented as the mean +/- S.E.M.; significance was calculated using a one-way ANOVA with a Tukey’s post-hoc test. There were no significant differences between the control lines (*Wt*^*PE*^ aka. *w*^*1118*^, *Wt*^*CS*^ aka. *CantonS*, *tinCΔ4Gal4*/+ and *UASseiRNAi*/+) for any of the measures. Significant differences were found between *sei* and *KCNQ* mutant lines compared to both wt controls as indicated (*p<0.05; ** p<0.01; ***p<0.001; ^%^p<0.05 compared to *Wt*^*PE*^ only). We also recorded from 3 week old *sei* KD hearts, *tinCΔ4Gal4*>*UASsei*RNAi, compared a outcrossed control lines (*tinCΔ4Gal4/+* and *UASseiRNAi*/+; ^#^p<0.05; ^##^p<0.01). N indicates the number of hearts providing 30 sec traces that were used for the analysis.

Interestingly, electrical activity in wt hearts changed with age, in that hearts from older (4–6 weeks) wt flies show triggered APs that are reminiscent of those seen in hearts from young *sei* mutants (Fig 4, compare B&D). This altered electrical activity in old wt hearts may reflect the age-related decline in repolarization reserve that we have previously documented [7,16]. Old *sei* and *KCNQ* mutant hearts almost always exhibited bursting APs, an exaggerated form of the electrical activity observed at younger ages (compare Fig 4B & 4E and 4C & 4F).

**Fig 5:**
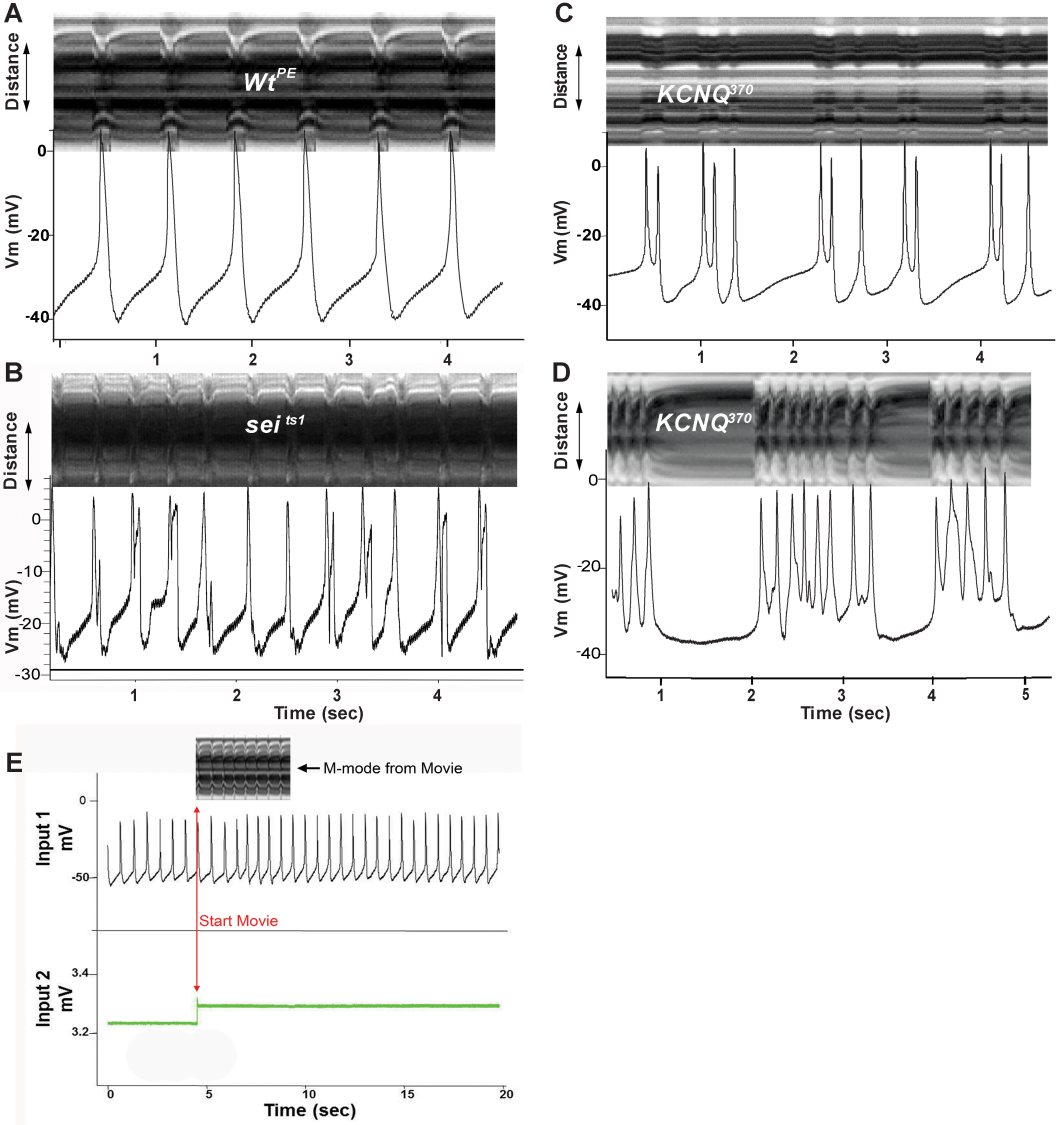
Simultaneous recording of heart wall movements and intracellular action potentials. M-mode traces (depicting heart wall movement in the y axis, top) were synchronized with intracellular recordings (showing spontaneous voltage changes, bottom) from the hearts of **(A)** a 1 week old *w*^*1118*^ wildtype fly **(B)** a 1 week old *sei*^*ts1*^ mutant **(C)** a 1 week old *KCNQ*^*370*^ mutant and **(D)** a 4 week old *KCNQ*^*370*^ mutant. **(E)** Simultaneous Intracellular / Optical Recording Setup-An intracellular recording from a single myocardial cell in the heart (Input 1) and the voltage trace generated by the optical recording software (Input 2) used to synchronize the optical and electrical recordings. An M-mode from the first four seconds of the movie is shown (top).

We wished to determine the relationship between the bursting electrical activity and cardiac contractions. To accomplish this hearts were not exposed to blebbistatin and we recorded intracellular electrical activity in spontaneously beating hearts while simultaneously recording high speed movies. Resting membrane potentials and electrical activity recorded under these conditions were similar to those observed with blebbistatin pretreatment (Fig 4A–4F), reaffriming that exposure to this compound did not significantly affect the electrophysiological activity and that for these short periods of electrical recording the impaled and beating myocardial cells were functioning as expected. In order to synchronize the electrical recording with the optical recording we had the image capture software send a signal to the amplifier that could be visualized in a separate channel (Fig 5E). Under these conditions we again observed regular, single peak action potentials in young wt hearts. Alignment of M-modes from the movies with the electrical activity that occurred during the filming interval showed that the action potentials in young wt hearts was correlated with individual heart contractions (Fig 5A). Electrical recordings from *sei* (Fig 5B) and *KCNQ* mutant hearts (Fig 5C & 5D) showed the expected triggered AP bursting activity and, notably, the AP peaks correspond 1:1 with the movements of the heart tube shown in the synchronized M-mode. Thus these AP bursts underlie the fibrillatory contraction patterns that we have previously documented in *KCNQ* mutants using our optical methodology [16].

### The selective hERG antagonist E4031 causes bradycardia in hearts from *wt* but not *sei* mutants

**Fig 6:**
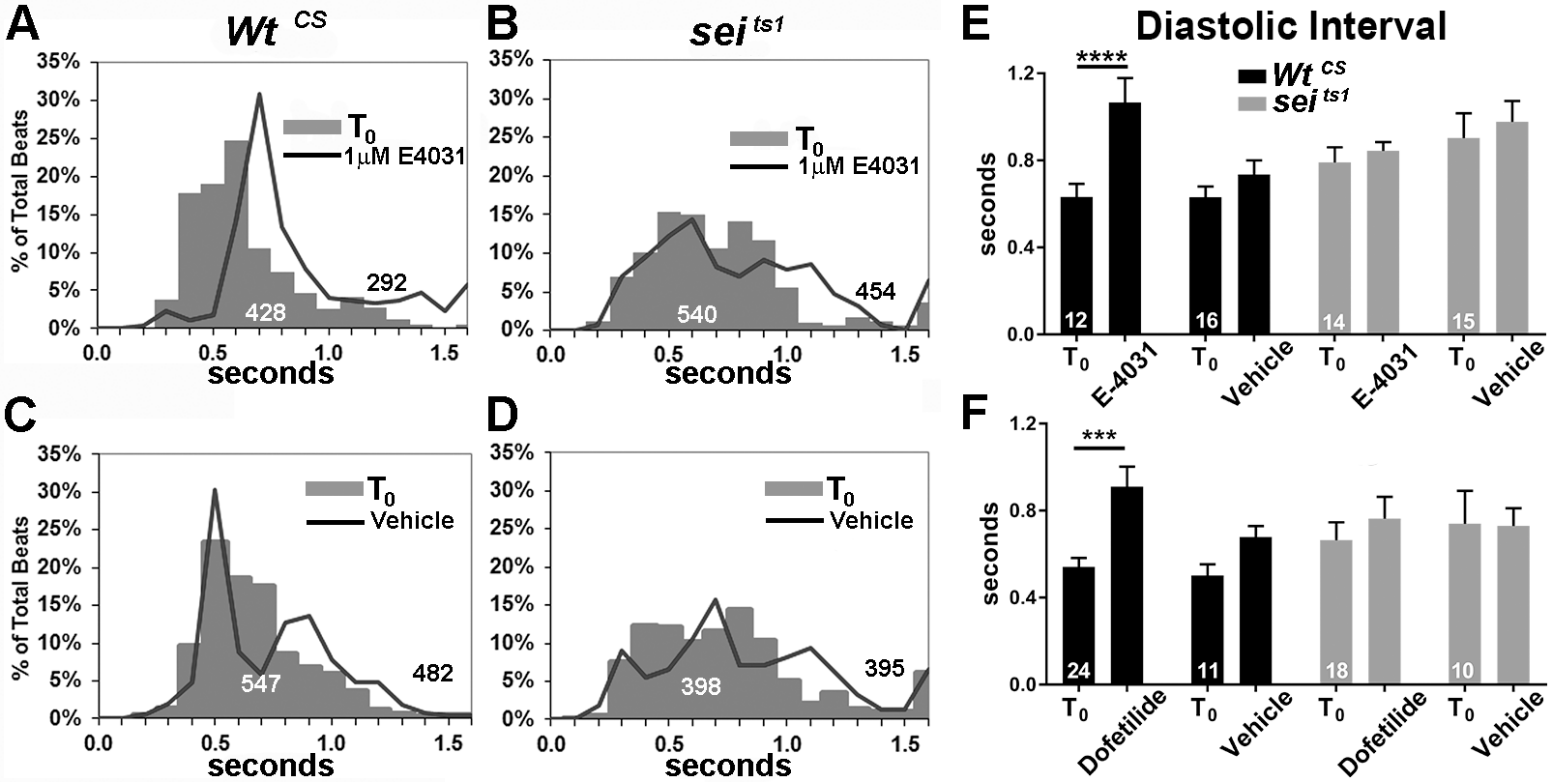
hERG antagonists E-4031 and Dofetilide cause bradycardia. **(A)** Histogram of binned diastolic intervals from 12 *Wt*^*CS*^ hearts before and after exposure to 1μM E-4031 expressed as a percentage of total DIs (totals are show in white for T_0_ and black for records at 15 min after drug / vehicle exposure). **(B)** Distribution of DIs in hearts from 14 sei/hERG mutants before and 15 min after exposure to 1μM E-4031. **(C)** Distribution of DIs in hearts from16 Wt^CS^ before and 15 min after exposure to AHL vehicle. **(D)** Distribution of DIs in hearts from 15 *sei/hERG* mutants before and 15 min after exposure to AHL vehicle. **(E)** Average DIs before and after exposure to either 1μM E-4031 or vehicle. **(F)** Average DIs before and after exposure to either 1μM Dofetilide or DMSO vehicle. Plotted values are mean ± SEM; significance was determined using a two way ANOVA and Sidak’s multiple comparisons test; *** p<0.001; ****p<0.0001.

We attempted to reproduce the effects of *sei* mutations using the selective hERG antagonist E-4031 [38]. Semi-intact heart preparations were recorded before and after a 15 m exposure to either AHL containing 1μM E-4031 or AHL alone (vehicle). Histograms showing the distribution of the individual DIs for all flies examined are shown in Fig 6. E-4031 caused a right shift in the DI peak from 0.6 to 0.7 sec as well as an increase in the percentage of DIs longer than 0.7 s in hearts from wt flies compared to baseline (T_0_, Fig 6A). Notably, there was a dramatic reduction in the total number of heart beats recorded in these 30sec movies in response to the drug exposure. In contrast the distribution of DIs in *sei* mutants is significantly broader at baseline than for *Wt*^*CS*^ and there is no shift in the maximum peak following drug exposure (Fig 6B). There is an increase in the percentage of DIs that are longer than 1.0 sec. Overall averages for all flies are shown in Fig 6E; *Wt*^*CS*^ hearts, but not *sei* mutants, exhibited a significant increase in DI following exposure to 1μM E-4031. A second, selective hERG antagonist Dofetilide [39] (at 1μM) produced similar bradycardia in hearts from wt flies, also characterized by significantly prolonged diastolic intervals and not observed in *sei* mutants (Fig 6F).

### Cardiac dilation and myofibrillar remodeling in *sei* but not *KCNQ* mutants

**Fig 7:**
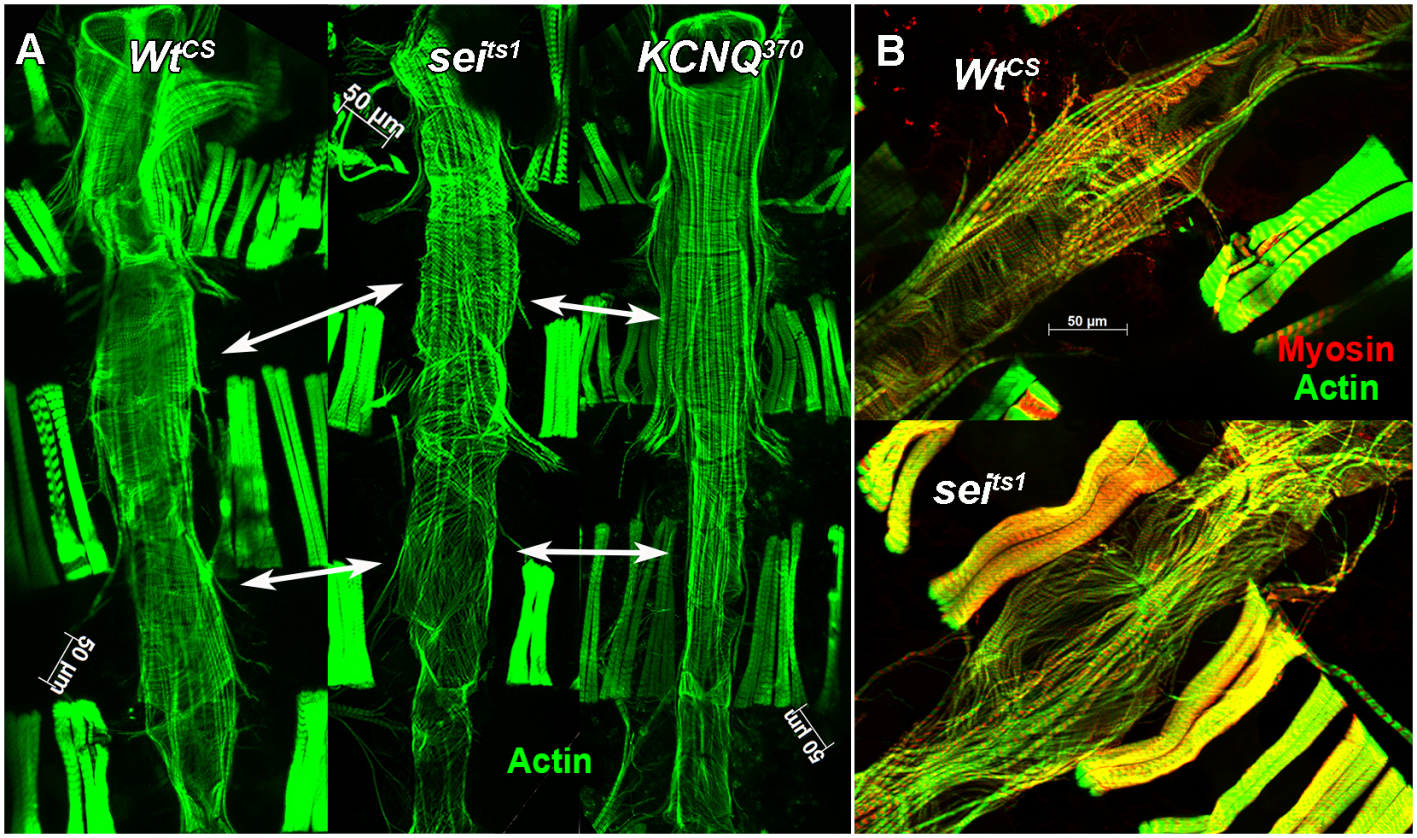
Actin and myosin staining of isolated hearts shows myofibrillar reorganization in *seizure* mutants. **(A)** Z stacks from *in situ* hearts (1 week old) stained with phalloidin to show actin filaments (green). Both *Wt*^*CS*^ and *KCNQ*^*370*^ mutant hearts show distinct circumferential myofibrils that are tightly packed. *Sei* mutant hearts show gaps between myofibrils that are disorganized (double headed arrows indicate comparable chambers of the heart). In addition, valve structures appear to be compromised (arrow heads). **(B)** Higher magnification of one chamber of the heart (abdominal segment 4) stained for actin (green) and myosin (red). Myofibrils in the heart from a 3 week old *Wt*^*CS*^ fly (top) are circumferentially organized and tightly packed; the same region from a 3 week old *sei*^*ts1*^ mutant heart (bottom) that shows severe myofibrillar disarray and obvious gaps.

To visualize muscle structure, hearts were exposed as for functional analyses and left *in situ*, attached to the dorsal cuticle. These hearts were then fixed and stained for muscle proteins as previously described [21,40]. Phalloidin staining of F-actin in hearts from wt flies reveals the distinctive circumferential organization of myofibrils within the myocardial cells that make up the heart tube. A similar circumferential arrangement was observed in the anterior-most region of *sei* mutant hearts, a structure called the conical chamber (Fig 7A, upper portion). However, myofibrils became increasingly thinner and this arrangement was more disorganized in more posterior chambers in *sei* mutant hearts with myofibrils oriented primarily longitudinally (Fig 7A, bottom set of double headed arrows).

**Fig 8:**
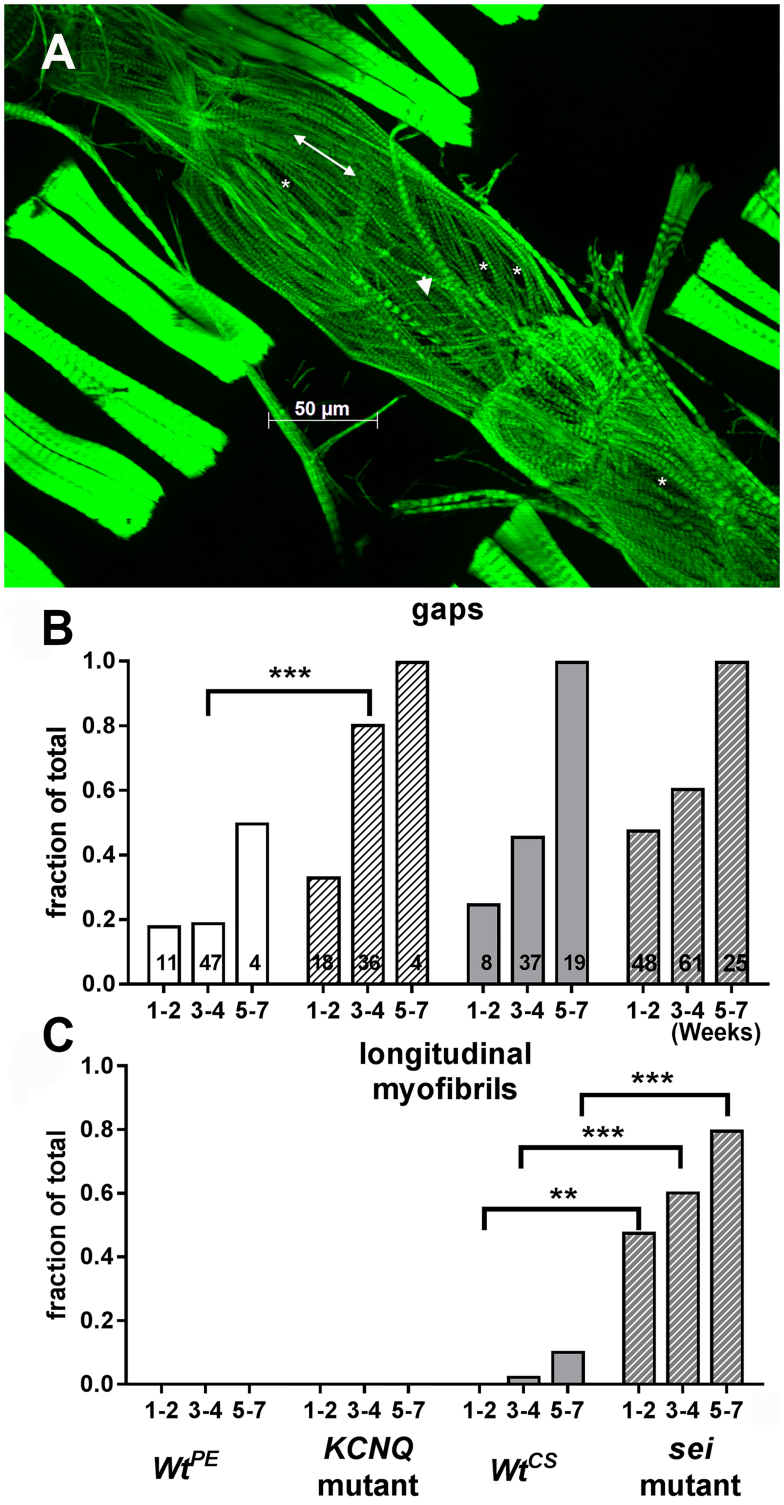
Morphological remodeling in seizure mutants. **(A)** A full projection Z stack of a heart from a 1 week old *sei* mutant stained with phalloidin for F-actin is shown. **(B)** Hearts were scored for presence of gaps (*in A) or **(C)** longitudinally oriented myofibrils (double headed arrow in A). Note also the presence of very thin myofibrils (arrow head in A, for wt structures see Fig 6B). Ages are in weeks; numbers of hearts examined are indicated in each bar in B. Significance was determined using multiple T-tests comparing each mutant with its genetic background control at each age; **p<0.01, ***p<0.001.

We examined hearts at different ages and found that this cardiac remodeling was age-dependent. We quantified the number of hearts showing structural alterations ranging from relatively mild (presence of gaps) to more severe (reoriented myofibrils) (Fig 8A). A relatively small percentage (20% or less) of hearts from young (1–2 week) wt flies (*Wt*^*PE*^ and *Wt*^*CS*^) showed gaps between myofibrils and none showed reorganization to a longitudinal orientation (Fig 8B & 8C). While almost 50% of hearts from young *sei* flies showed gaps, this was not significantly different from the *Wt*^*CS*^ control. However, *sei* mutant hearts did exhibit significant reorganization of their myofibrillar structure at 1–2 weeks of age. By 5–7 weeks 80% of the hearts from *sei* mutant flies showed this more severe form of myofibrillar remodeling (Fig 8C). In contrast only the CS background controls showed evidence of structural rearrangement in older flies (Fig 8C). The reorganization of circumferential to more longitudinally oriented disorganized myofibrils was most reliably observed in the heart chamber located in the fourth abdominal segment (compare Fig 7B & 7C). This correlates very well with our observations that many hearts from older flies showed abnormal contractions in this same region (see S1 Movie).

**Fig 9:**
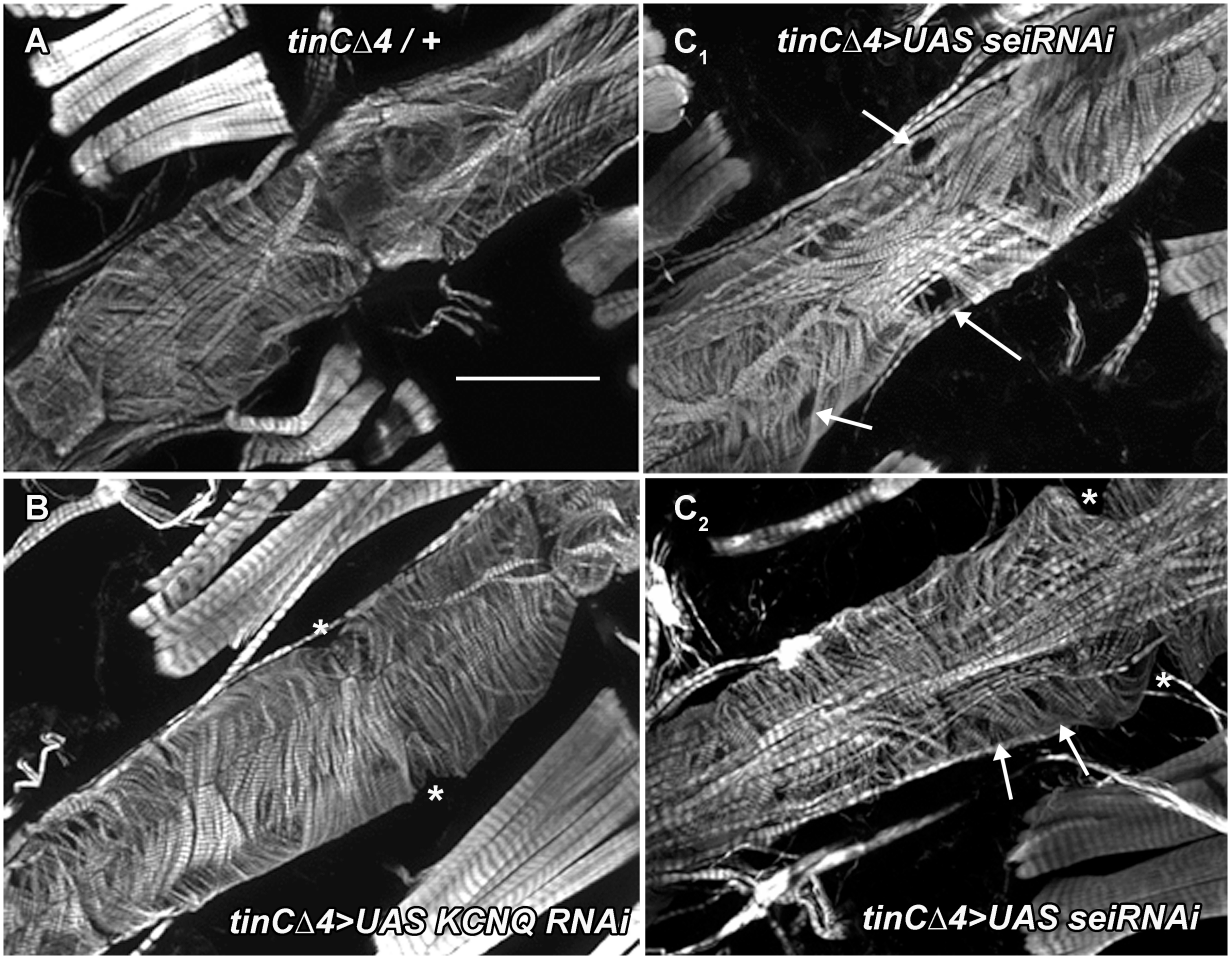
Cardiac-specific *sei* KD causes myofibrillar disarray and thinning. **(A)** Phalloidin staining of F-actin in hearts from 3 week old flies reveals the circumferential myofibrillar structure in control (*tinCΔ4Gal4*/+). Scale bar is 50μm. **(B)** Hearts for 3 week old KCNQ KD flies (*tinCΔ4Gal4>UASKCNQ RNAi)* also show a normal circumferential myofibrillar pattern. **(C)** Cardiac-specific KD of *sei* results in myofibrillar disarray and gaps (arrows). * denotes the position of ostia.

To establish that this remodeling was cardiac autonomous we used *tinCΔ4-Gal4* driver to express either *UASsei RNAi* or *UAS KCNQ RNAi* specifically in myocardial cells during embryonic and adult (but not larval) stages. 3-week old adult hearts from outcrossed driver flies showed the expected circumferential arrangement (*tinCΔ4-Gal4*/+, Fig 9). However, cardiac *sei* KD showed evidence of gaps (*tinCΔ4-Gal4*>*UASsei RNAi*, Fig 9C_1_ & 9C_2_). Myofibrillar structure in the *KCNQ* KD hearts more closely resembled that of the controls (Fig 9B). These finding are consistent with data from our functional analysis of hearts with *sei* KD, which revealed a significant reduction in contractility (Fig 1H).

**Fig 10:**
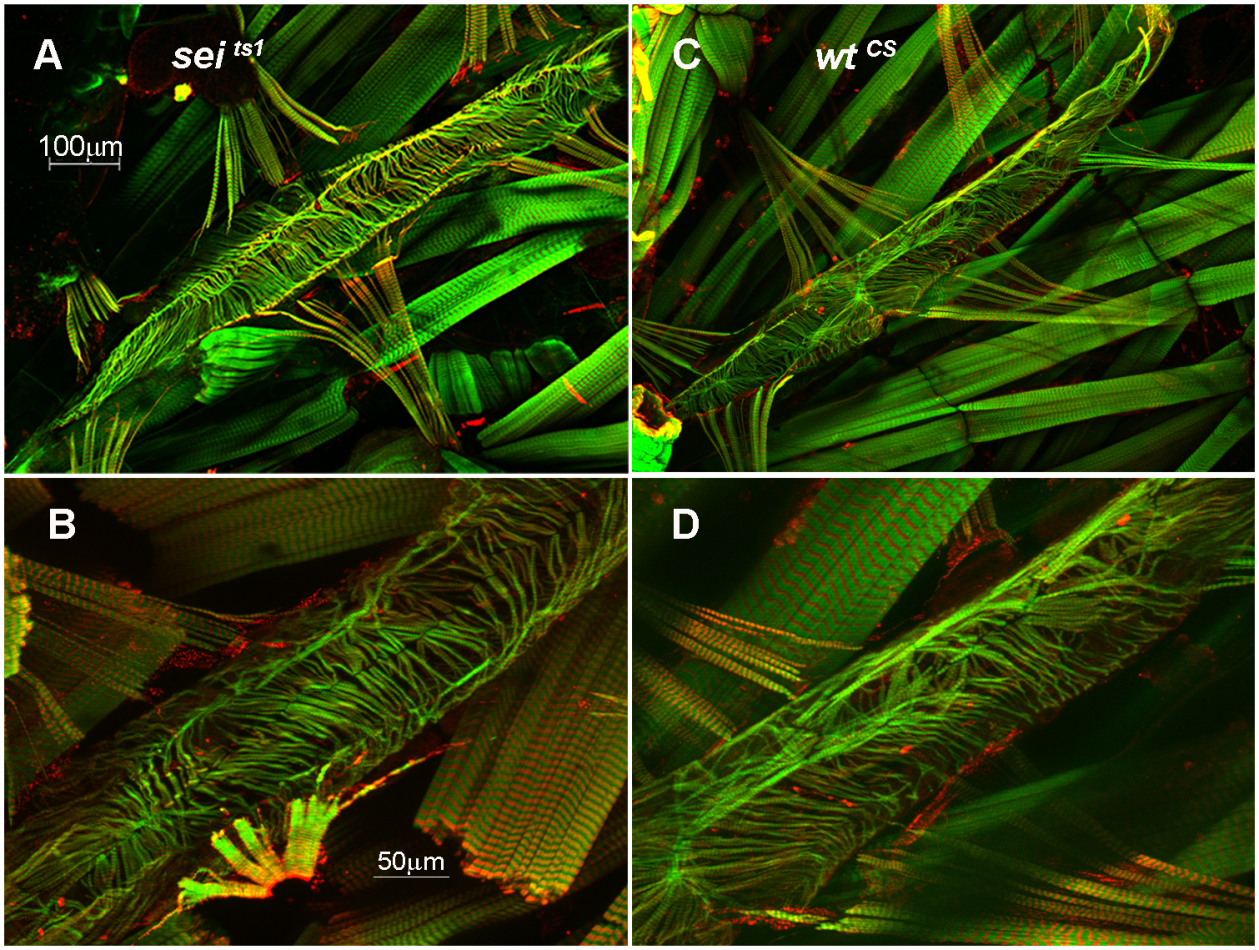
Larval heart morphology in *sei* mutants. Hearts from wandering 3^rd^ instar larva (just prior to pupation) were fixed and stained for F-actin (green) and myosin (red). The posterior abdominal heart is shown for a *Wt*^*CS*^ **(A&B)** and *sei*^*ts1*^ mutant **(C&D)** heart at 10x (top) and 25x (bottom). No obvious differences were observed for mutant hearts compared to *wt* at this late larval stage.

Since it is possible that the remodeling we observed was due to a developmental defect we examined heart structure in 3^rd^ instar larva just prior to pupation. The myofibrillar organization in wt wandering third instar larva revealed a more loosely organized arrangement compared to adults. However we observed no gross differences in size or in the overall arrangement of myofibrils between *wt* and *sei* mutants (Fig 10A–10D).

### Expression analysis of *sei* and *KCNQ* mutant hearts

**Fig 11:**
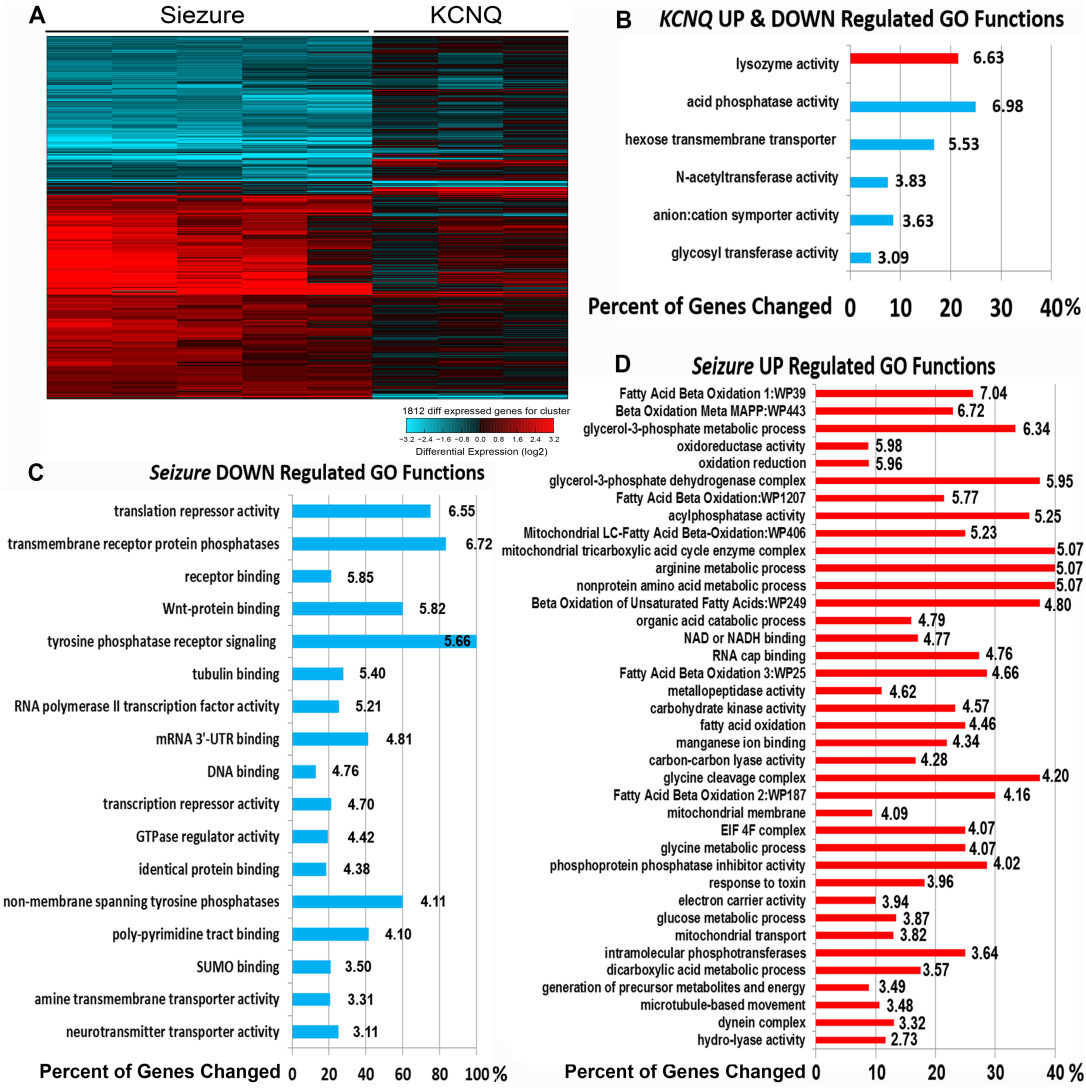
Altered gene expression and GO Functions in *Sei* and *KCNQ* mutant hearts. **(A)** Isolated hearts from control and channel mutant flies were subjected to gene expression analysis. Heat map shows hierarchical clustering of 1812 probesets that were significantly up (red) or down (blue) regulated in *sei* mutant hearts compared to *Wt*^*CS*^ (Fold>2.0, P<0.05, each line shows results for a single probeset and each column is a replicate) and the corresponding effect in hearts from *KCNQ* mutants (compared to *Wt*^*PE*^). **(B)** Significantly UP (red) and DOWN (blue) regulated Go Functions (Z score>2) identified in *KCNQ*^*370*^ mutant hearts are shown with the corresponding percent of category genes that were significantly altered. Z scores are shown at the end of all bars. **(C)** Significantly Down regulated Go Functions identified in *sei* mutant hearts. **(D)** Significantly UP regulated Go Functions (Z score>2) in *sei* mutant hearts are shown.

We used Affymetrix microarrays to analyze gene expression in *KCNQ* (N = 3 replicates) and *sei* (N = 5 replicates) mutants as well as their respective background controls; *CS* (N = 5 replicates) and *KCNQ*^*97*^ (N = 3 replicates). Interestingly, we found a dramatic difference between the two mutants in the numbers of misregulated genes, with sei mutants having significantly more genes with altered expression than KCNQ mutants. We identified 1019 upregulated and 712 downregulated probesets in *sei* mutants compared to controls, but only 91 upregulated and 68 down-regulated probesets in *KCNQ* mutants compared to controls (|Fold|>2 and *P*<0.05) (Fig 11A, S1 Dataset).

Gene set enrichment analysis of *sei* mutant data showed 6 GO function categories were significantly (Z score >2) altered in *KCNQ* mutant hearts compared to background controls (Fig 11B) compared to significant 17 down regulated and 38 up regulated GO function categories in the *sei* mutant hearts (Fig 11C). The top down regulated functions included “repression of translation”, “protein phosphatases”, “receptor binding” and “wnt protein binding”. The majority of the up regulated functional categories (32/38) were involved in some aspect of cell metabolism. Consistent with the morphological data, a number of sarcomere-related proteins were also significantly down regulated (p<0.05) in *sei* but not *KCNQ* mutant hearts, although most of the |Fold| changes were less than 2. Nevertheless we were able to confirm a significant downregulation of *MHC* das well as *adenomatous polyposis coli* (*APC*), a Wnt pathway gene with qPCR (S2 Fig).

### *sei* interacts genetically with Wnt effector *pygopus* in the fly heart

Our previous studies revealed a required role for the Wnt signaling gene *pygopus* (*pygo*), that codes for a β-catenin-associated nuclear receptor protein[41–44] in heart function [45,46]. These studies showed that *pygo* KD in the heart caused bradycardia and myofibrillar remodeling associated with a reduction in contractility, phenotypes that are reminiscent of the effects shown above for *sei* mutant hearts. In addition, our microarray analysis suggests that *pygo* expression may be downregulated in *sei*, but not *KCNQ*, mutant hearts (S3 Dataset).

**Fig 12:**
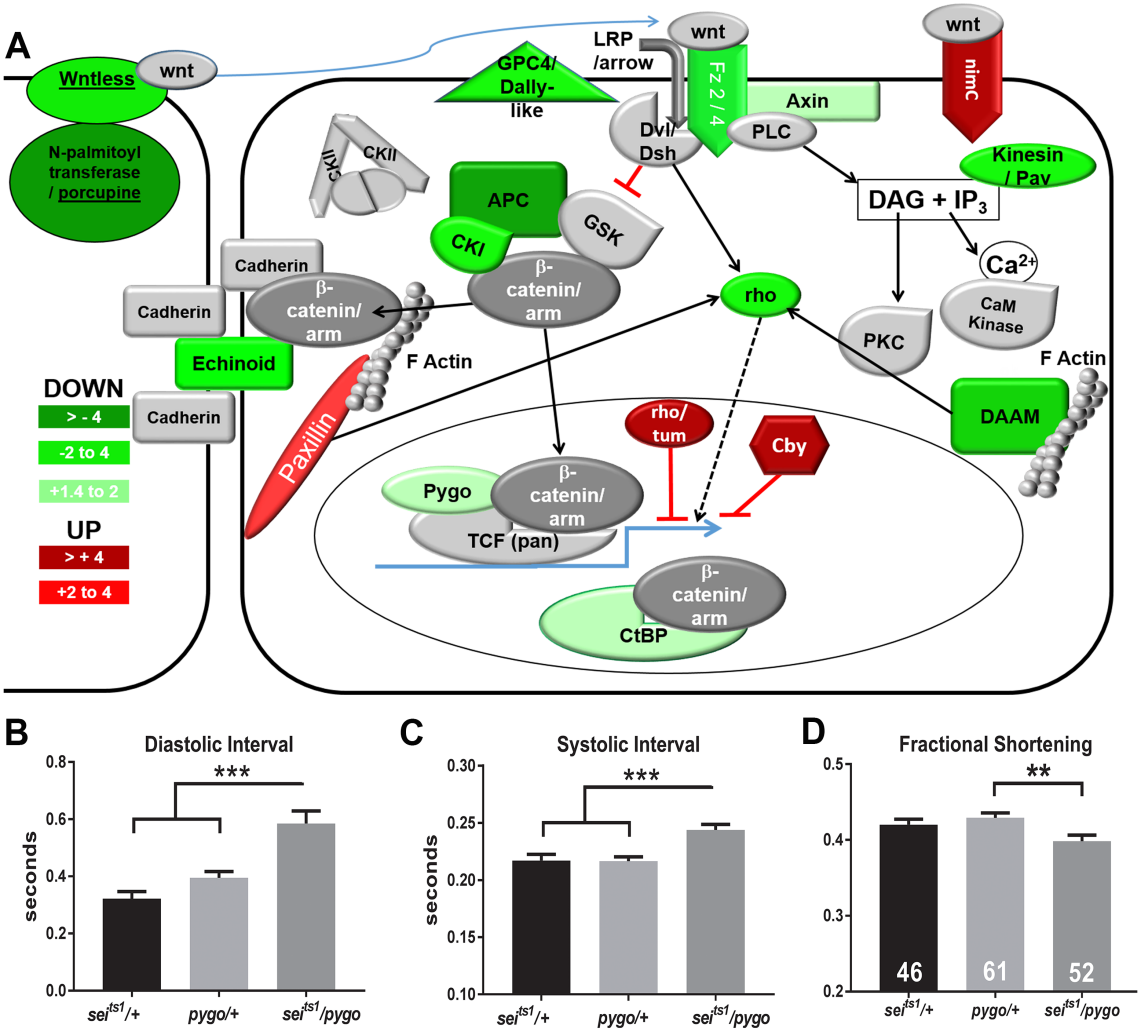
*sei* mutations affect *wnt* gene expression and interact genetically with pygo in the heart. **(A)** Schematic diagram of the Wnt signaling pathway highlighting the individual components showing significant down (green) or up (red) regulation in hearts from *sei* mutants compared to its *Wt*^*CS*^ background control. **(B)** Diastolic intervals in *sei*^*ts1*^*/ pygo* double heterozygotes are significantly longer than for *sei*^*ts1*^ and *pygo* single heterozygotes. **(C)** Systolic intervals in *sei*^*ts1*^*/ pygo* double heterozygotes are significantly longer than for *sei*^*ts1*^ and *pygo* single heterozygotes. **(D)** Fractional shortening, a measure of contractility, is significantly reduced in *sei*^*ts1*^*/ pygo* double heterozygotes compared to *sei*^*ts1*^ and *pygo* single heterozygotes. Significance was determined by one way ANOVA and Tukey’s post-hoc test; **p<0.01, ***p<0.001.

We used a double heterozygote approach to look for genetic interactions between *pygo* and *sei*. We examined cardiac function in single heterozygotes of *sei* and *pygo* mutants (*sei*^*ts1*^/+ and *pygo*/+). For either single heterozygote, the mean diastolic and systolic intervals were not significantly different from each other (Fig 12B) and were similar to those seen for controls (Figs 1 & 2). In contrast, we observed significant bradycardia for the double heterozygote combination (*sei*^*ts1*^*/pygo*, Fig 12B). Fractional shortening was also significantly reduced in the *sei*^*ts1*^*/pygo* double heterozygotes compared hearts from single heterozygotes (Fig 12D). Taken together these data suggest a genetic interaction between the *sei* K^+^ channel gene and Wnt signaling.

## Discussion

Channel dysfunction, or channelopathies, underlie a number of cardiac disorders such as LQTS and are thought to contribute to sudden cardiac arrest, infant sudden death syndrome [47] and increased risk of cardiac arrhythmias. The human ether-a-go-go related K^+^ channel (hERG) along with the *KCNQ* K^+^ channel (I_Kr_, and I_Ks_ respectively) are the major contributors to cardiac repolarization (Phase 2 & 3) in humans. Defects in KCNQ and hERG channels have been shown to cause LQT1 and LQT2, respectively and cardiac arrhythmias in humans. The hERG channel in particular has been a major target for the development of anti-arrhythmia drugs and can be inhibited by a variety of drugs that do not specifically target the heart [48,49]. We have previously shown that the *KCNQ* K^+^ channel is functional in the adult fly heart and that mutations in this channel contribute to cardiac arrhythmias [16]. In the fly, *seizure* and *KCNQ* mutations do not significantly affect cardiac development (Fig 6), although in the mouse some *hERG* mutations cause looping defects and embryonic lethality [50]. Importantly for heart function studies, survivorship of adult flies is not acutely affected by badly functioning hearts that would quickly result in death in vertebrates, likely because the fly does not rely on the heart for oxygen distribution, which is carried out by a separate system of tracheoles.

The current data demonstrate that the *sei* gene, the fly homolog of *hERG*, along with a number of other genes encoding K^+^ channels, are also expressed in fly myocardial cells (Fig 1A) and likely contribute to the repolarization capacity of the heart. This set of K^+^ channels is reminiscent of what is observed in vertebrate hearts [1] [2]. The primary effect of *sei* dysfunction in mutants was bradycardia and this phenotype could be replicated by cardiac-specific and adult, cardiac-specific *sei* KD (Fig 2) as well as acute application of selective hERG antagonists (Fig 6). However, the increases in SI seen in both KD experiments (Fig 2) are different from the mutant phenotype and possibly reflect some compensatory genetic alterations in the systemic *sei* mutants. Our data also show that, as in humans, mutations in *sei* cause arrhythmias (Fig 1E) and electrical remodeling in the form of AP bursts that are likely triggered by EADs (Figs 4 and 5, quantified in 6). In addition we show that systemic mutants or cardiac-specific KD of *sei*, but not of *KCNQ*, reduced heart contractility (Figs 1G and 2D), and were associated with structural remodeling (Figs 7 and 8).

These effects appear to be specific to alterations in *sei* as most of the *sei* cardiac function phenotypes can be rescued by over-expression of the wt channel (Fig 1). In addition, the morphological/functional effects of channel dysfunction appear not to be due to developmental defects (Fig 10) but are adult stage- and cardiac-specific (Figs 2 and 9).

The intracellular electrical activity in adult fly cardiomyocytes appears more nodal or atrial-like [1,2] and is similar to what has previously been observed in larva [35,36,51] although more robust in terms of the resting potential and AP amplitude (Table 1, Figs 4 & 5). We observed frequent AP bursts in both *sei* and *KCNQ* mutants and in hearts from old flies compared to young Wt fly hearts (Figs 4 and 5) reminiscent of the increase in electrical remodeling with disease and age in human myocardial cells [52,53]. However, there appears to be a significant difference between mutations in *KCNQ* and *sei* in terms of their effects on electrical and morphological remodeling, with mutations in *KCNQ* resulting in significantly more electrical arrhythmia at younger ages compared to *sei* mutations (Figs 4, 5 and 6). In addition, although 100% of APs recorded from both old *KCNQ* and *sei* mutants are arrhythmogenic, the severity of the events appears to be worse in *KCNQ* mutants. Notably, our ability to simultaneously record both intracellular APs while optically monitoring intact heart function demonstrate that the arrhythmogenic AP’s we observe correlate directly with unsustained fibrillatory contractions of the heart wall revealed in M-modes (Fig 5).

Although both *sei* and *KCNQ* channel mutations cause electrical remodeling and arrhythmia in the fly heart model they appear to have differing effects on muscle structure (Fig 7). Mutations in *sei* appear to be linked to an increase in myofibrillar disorganization (Fig 7) and this effect on myocardial structure is manifest by reductions in fractional shortening (Fig 1H). In addition, the observed reductions in shortening velocities under loaded conditions suggests that the ability to generate tension is significantly lower in hearts from *sei* mutants than for controls or *KCNQ* mutants and are consistent with the reduced ability to sustain an isometric contraction in the *sei* mutant hearts (Fig 3). Together these results suggest differential roles for these channels on structural and electrical integrity of the adult heart.

Our microarray results suggest that the different effects of the two K^+^ channel mutants are the result of underlying differences in gene expression. Our observation that many pathways involved in metabolism are significantly upregulated in *sei* mutants is consistent with previous observations in rabbits, although in that study both LQT1 and LQT2[54] models exhibited similar effects. In particular, in the adult fly heart the Wnt signaling pathway appears to be selectively perturbed in the *sei* mutant hearts, as evidenced by a downregulation of many of its pathway components. This is consistent with our previous results demonstrating that mutation or cardiac KD of the TCF co-factor encoded by *pygo* [45] cause bradycardia and morphological remodeling similar to that observed for *sei* mutants. Importantly, we now demonstrate an interaction between *sei* channel mutations and mutations in *pygo* (Fig 11B–11D). Wnt signaling has previously been shown to play significant roles in cardiac development in flies[55–58] and vertebrates[59,60] and in cardiac disease [59]. We have shown in the fly that *pygo* likely plays a role in the maintenance of cardiac function and structure in the adult [45,46] and our current data suggest that Pygo and Sei channel dysfunction are likely linked genetically. Many components of both the canonical and non-canonical pathways exhibit reduced expression, which would suggest an overall reduction in Wnt signaling. However, we also observe downregulation of potential negative effectors of Wnt signaling, such as APC, the core of the destruction complex, and CtBP, which has been shown to mediate both activation and repression of transcription[61]. Both effects could be expected to result in increased stabilization of β-catenin. Thus, the exact role of different Wnt signaling components in maintaining cardiac structure and function remains to be determined.

Electrical and morphological remodeling have previously been shown to be linked; for example, knockout of the heart development transcription factor Ptx1 was shown to affect both electrical and morphological remodeling in mouse and humans [62]. A number of studies have documented pro-fibrotic and apoptotic effects of atrial fibrillation [63–67] as well as tachypacing in cardiomyocytes[68], in dog models[69] and in response to sustained atrial fibrillation in patients[63]. Further, a genetic variant situated close to the long QT syndrome (LQTS) type 2 gene *KCNH2* has been shown to be associated with early onset AF [70]. Thus, cellular/molecular links between channel function, electrical activity and morphological remodeling and ultimately heart failure have been suggested but have yet to be clearly elucidated [66]. An understanding of how hERG channels interact with cellular pathways involved in electrical and structural remodeling and with other repolarizing currents such as I_Ks_, mediated by KCNQ channels, will be important to the development of novel anti-arrhythmia therapies. Our data using the fly heart model now provides the first clear genetic and physiological evidence that some channelopathies may be contributing to cardiac remodeling during disease progression via Wnt signaling.

## Methods

### *Drosophila* strains

Adult flies were collected upon eclosion, segregated by sex and raised at 25°C with food changes every 3 days. *Sei* mutants, *sei*^*ts1*^ and *sei*^*ts2*^, were previously isolated in a screen for ethylmethane sulfonate (EMS)-induced temperature-sensitive paralytic mutations on the second chromosome [71,72] and were a gift from Dr. Barry Ganetzky. *Sei*^*ts1*^ mutation introduces a stop codon before the membrane spanning region and *sei*^*ts2*^ has a Glu-to-Lys substitution at a critically conserved site at the channel pore [70]. The *KCNQ*^*370*^ mutants were created by imprecise P-element excision that also introduces a stop codon before the membrane spanning region as previously described [16]. We used the genetic background strains of these mutations as Wt controls: *Canton S* (designated *Wt*^*CS*^, Bloomington Stock Center) for the *sei* mutants and a line with a precisely excised P element from the KCNQ locus of *w*^*1118*^ flies (designated *Wt*^*PE*^, see [15]) for the *KCNQ* mutants. For knockdown experiment we used *UAS-seiRNAi* line (VDRC #190504), *UAS-KCNQ RNAi* (VDRC #106655) and *UAS-APC2 RNAi* (VDRC #100104) from the Vienna Drosophila Resource Center (Vienna, Austria). Heart-specific driver line used was *tinCΔ4-Gal4* [73] that express in the myocardial cells of the heart. A temperature sensitive *tubulin-Gal80*^*ts*^*Gal4* driver that expresses Gal4 only when shifted to permissive temperatures above 18°C was used in combination with *tinCΔ4-Gal4* and UAS-*sei* RNAi to specifically KD *sei* in the adult heart [74,75].

### BAC genomic rescue of *Seizure*

*Seizure* rescue flies (*Sei+*) were generated using PBac {yellow[+]-attP-3BVK00033 (cytological region 65B2 on 3rd chromosome; strain identifier at BestGene: 9750). A 21-kb BAC (#CH322-81K08) in the attB-P[acman]-CmR-BW [76] plasmid, which contains genomic DNA that spans the *seizure* locus, was obtained from the BACPAC Resources Center. The genomic DNA was introduced at the VK33 landing site [76], on the third chromosome by BestGene. The *sei*^+^ transgenic flies were verified by PCR and eye color. *Sei*^+^ was crossed into the *seizure*^*ts1*^ mutant [72] that had itself been previously recombined into a *yw* background similar to the rescue fly’s. The crosses yielded y[1], *w[1118] /w[1118]; sei*^*ts1*^/ *sei*^*ts1*^; *sei*^+^/*sei*^+^ which was used as the mutant rescue strain and w^-^/w^-^; *sei*^*ts1*^/*sei*^*ts1*^;+/+ used as the control.

### Semi-intact preparations and Semi-automated Optical Heartbeat Analysis (SOHA)

Hearts from adult flies were exposed by dissection in room temperature bubbled artificial hemolymph (AHL, [77]). Heart movements were filmed with a Hamamatsu EM-CCD camera and recorded using HC Image software (Hamamatsu Corp). Heartbeat movements were analyzed as previously described using SOHA software ([16,31,77,78]; http://sohasoftware.com/). For some experiments heart function was examined under loaded conditions as previously described (Cammarato et al, 2015). Semi-intact preparations were first filmed in artificial hemolymph which was then replaced with AHL containing 20% Ficoll 400 (w:v, Sigma Aldrich). Hearts were incubated with aeration for 30 min in this solution and filmed; following filming the Ficoll solution was replaced by AHL and hearts were allowed 30 min to recover before being filmed a final time. For these latter experiments frame rates were 200–250 fps.

### Adult *Drosophila* cardiomyocyte electrophysiology

The semi-intact heart dissections and optical recording was performed as described above. The semi-intact preparation was incubated in artificial hemolymph containing 10 μM blebbistatin (Sigma Aldrich) and equilibrated with oxygenation in the dark for at 45–60 minutes until the hearts stopped beating[33,34]. The preparation was then supplied with fresh saline without blebbistatin and electrical potentials were recorded from the conical chamber using sharp glass electrodes (20–50 MΩ) filled with 3M KCl and standard intracellular electrophysiological techniques. Data were acquired using an Axon-700B Multiclamp amplifier, signals were digitized using the DIGIDATA 1322A and data were captured and analyzed using PClamp 9.0 and Clampfit 10.0 software respectively (all from Molecular Devices). Data was quantified from representative 30s recordings where the resting membrane potential had remained stable for at least 30s. APD10, 50 and 90 data was determined for 20–50 individual APs per fly and averaged.

### Simultaneous optical and electrophysiological recordings

For simultaneous recordings we again used the semi-intact heart dissection as described above, however, we did not treat the prep with blebbistatin, thus it continued to actively beat during impalement and electrical recording. To coordinate the optical and electrical recordings a TTL pulse was sent by the image capture software to the Digitizer. The pulse duration lasted for the entire period of optical recording and was recorded in a separate channel by the PClamp software allowing us to delineate the beginning and the end of the optically recording directly within the electrical record (Fig 4).

### Pharmacology

E-4031 and Dofetilide were obtained from Sigma-Aldrich. E-4031 stock solution (10 mM) was prepared by dissolving in distilled H_2_O; Dofetilide was dissolved in DMSO to make a 10mM stock. Hearts were dissected from both *Wt*^*CS*^ and *sei*^*ts1*^ mutant flies, allowed to equilibrate for 30 min in oxygenated AHL and were then filmed for 30sec (T_0_). The hemolymph was replaced with AHL containing either 1μM E-4031 or 1 μM Dofetilide. For E4032 experiments a second set of hearts were filmed using AHL as the “vehicle” and for Dofetilide an equivalent amount of DMSO without drug was added to the AHL. Hearts were filmed again for 30sec following a 15 min exposure to either drug or vehicle.

### Immunostaining of adult *Drosophila* hearts

Semi-intact adult heart preps were prepared as above and larval hearts were dissected as previously described [79]. In all case hearts were stained as described in [40]. Briefly, hearts were fixed in 4% paraformaldehyde/phosphate-buffered saline (PBS) for 20 min, washed three times in PBT (PBS/0.1% Triton X-100) and incubated with primary antibodies in PBT for 2 hours. Hearts were then washed (3x 10’) incubated with secondary antibodies and phalloidin for 1–2 hours at room temperature. After washing in PBT (3x 10’), they were mounted onto slides in Vectashield mounting medium for Florescence with DAPI (Vector Labs) and analyzed using ApoTome (Carl Zeiss). The monoclonal antibody against Pericardin was obtained from the Developmental Studies Hybridoma Bank developed under the auspices of the NICHD and maintained by The University of Iowa, Department of Biology, Iowa City, IA 52242. Antibodies against *Drosophila* Myosin were a gift from Dr. G. Melkani [80]. The secondary antibodies used were anti-rabbit and anti-mouse conjugated with Alexa Fluor 555 dye and Alexa Fluor 488 (Molecular Probes, Eugene, OR, USA).

### RT-PCR

Total RNA was extracted from 10 adult hearts using TRIzol (Invitrogen, Carlsbad, CA) and purified with Mini RNA isolation kit (Zymo Research, Orange, CA) for heart RNA. After treatment with DNase I, first strand cDNA was transcribed by SuperScript III (Invitrogen) by oligo(dT)12-18primer, followed by second-strand synthesis. RT-PCR was carried out by using LightCycler FastStart DNA Master PLUS SYBR Green I kit (Roche, Basel, Switzerland). Semi-quantitative PCR was performed for 32 cycles that consisted of denaturation at 94°C for 30 s, annealing at 65°C for 30 s, and extension at 72°C for 30 s by using platinum Taq DNA polymerase (Invitrogen).

To verify the microarray results we performed qPCR analysis on 3 biological replicates each containing 10–12 adult hearts from both Wt^CS^ and sei^ts1^ mutants. RNA was extracted using QIAzol (QIAGEN, Ref# 79306) and purified using miRNeasy Mini Kit (QIAGEN, Ref# 217004) following manufacturer’s instructions for RNA purification of small biological sample sizes. Genomic DNA removal and cDNA synthesis was done using Quantitec Reverse Transcriptase kit (QIAGEN, Ref# 205311; see S1 Table for primer sequences). Quantitative PCRs were carried out with the LightCycler 96 Instrument (Roche, Ref# 05815916001) and FastStart Essential DNA Green Master kit (Roche, Ref# 06402712001) was used for SYBR Green I-based real-time PCR. Amplification was performed for 35 cycles that consisted of denaturation at 95°C for 15 s, annealing at 60°C for 15 s, and extension at 72°C for 10 s (see S1 Table for primer sequences). Delta Delta CT (cycle threshold) values were calculated by first normalizing the CT values for each target gene to the average CT value for the actin (reference) gene and then normalizing the Δ CT for each experimental gene in *sei* hearts to the average ΔCT score from *wt*^*CS*^ control hearts.

### Fluidigm analysis

5 fly hearts were isolated and snap frozen in 10μl of water. Each heart was lyophilized and lysed for 2 minutes at 98°C in lysis buffer (LB) (0.25% NP40 in H_2_O). The lysed hearts were then briefly centrifuged, and 30μl supernatant transferred to a fresh tube. Reverse transcription was carried out by using 3.3μl of lysed heart (~8% of a single heart) in a 5μl final reaction volume using the VILO Reaction mix as per the manufacturer’s instructions (Life Technologies). Genes were pre-amplified using a Fluidigm Master Mix (Fluidigm). For removal of single stranded DNA prior to nanofluidic cycling, 6 μL of Exosap solution (4.2 μL of water, 0.6 μL of Exonuclease 1 Rn Buffer, Exonuclease 1 (20 units μL^−1^, New England Biolabs) was added to the 15 μL final reaction volume of the RT step. The resulting 21 μL final volume was then incubated at 37°C for 30 min and then heat inactivated at 80°C for 15 min. The pre-amplified volume (21 μL) was then diluted 10-fold in DNA suspension buffer (Teknova), and stored at −20°C prior to running on the chip. Pre-amplified products for each individual fly heart was then assayed using Fluidigm's 48 nanofluidic qPCR arrays on a Biomark system (Fluidigm), according to their protocols. Biotium's EvaGreen DNA binding dye was used to detect amplified product according to Fluidigm's protocols. Values were calculated taking the experimental gene raw Ct values minus concomitant housekeeping control gene (minichromosome maintenance complex component 2, MCM2). Delta Ct values were calculated relative to raw Ct values from the wildtype Canton-S (Wt^CS^) fly line. Ct values are inversely correlated with relative expression (see S1 Table for primer sequences).

### Microarray and bioinformatics analyses

We used GeneChip Drosophila Genome 2.0 Array to analyze gene expression for microarrays (30, 3-week old hearts per array) for each mutant line: *sei*^*ts1*^ (N = 5 replicates) and *KCNQ*^*370*^ (N = 3 replicates) and for control flies representing each genetic background, *Canton-S* (*CS*; N = 5 replicates) for the *sei* mutants and the precise excision control (*KCNQ*^***97***;^ N = 3 replicates) for the *KCNQ* mutants. Affymetrix.cel files were quality controlled with the Bioconductor package affyQCReport [81]with R, an open-source software environment for statistical computing and graphics. RMA normalization for the dataset was then conducted with AltAnalyze [82,83]. For clustering analysis differentially regulated genes where considered those with an absolute fold change greater than 2 and a P<0.05 in compared to each mutants control line (1812 probesets). Clustering was conducted in AltAnalyze using the HOPACH algorithm with cosign similarity. For Gene Ontology (GO) enrichment analysis, GO-Elite [84] was used to find the enrichment of GO terms amongst the significantly up and downregulated genes (|Fold|>2, P<0.05) using each control stain as a base line. Raw data files and RMA normalized expression matrix have been deposited at GEO (GSE94589).

## Supporting information

S1 Fig Heart function parameters with age in *sei* mutants. **(A)** Mean diastolic intervals are significantly increased at all ages in hearts from *sei* mutants. **(B)** Mean systolic interval lengths are not significantly affected in *sei* mutants. **(C)** Arrhythmia is significantly increased in *sei* mutants at young ages although the increased arrhythmia at old ages in mutants was not significantly different from *Wt*^*CS*^. **(D)** Hearts from female *sei* mutants are significantly smaller during diastole that controls at all ages. **(E)** Systolic diameters did not show consistent differences in hearts from *sei* mutants. **(F)** Cardiac contractility, measured as fractional shortening, is significantly reduced at all ages in *sei* mutants.
(TIF)

S2 Fig Relative gene expression change in isolated fly hearts. 10–12 pooled isolated hearts from both *Wt*^*CS*^ and *set*^*ts1*^ mutants were subjected to qPCR analysis for actin, myosin heavy chain and APC. ΔΔ CT values were calculated relative to actin for each sample (triplicate experimental replicates and triplicate biological replicates, significance calculated by unpaired t-test, *p,0.05, **p,0.01). Note that Ct values are inversely correlated with relative expression.
(TIF)

S1 Movie Comparison of *Wt* and *sei* mutant heart function. 15 second video clips of *Wt*^*CS*^ heart (top), a representative *sei*^*ts1*^ mutant heart (middle) and a sei^ts1^ mutant heart with an extreme case of contractile dysfunction (bottom). All flies were 1 week old.
(MP4)

S1 Dataset Gene expression data set for *sei*^*ts1*^ v. *Wt*^*CS*^ and *KCNQ*^*370*^ v. *WtPE*. (XLSX)

S1 Table List of primers. (DOCX)

## References

[1] Nerbonne JM (2004) Studying cardiac arrhythmias in the mouse—a reasonable model for probing mechanisms? Trends Cardiovasc Med 14: 83–93. 10.1016/j.tcm.2003.12.006 15121155

[2] Nerbonne JM, Kass RS (2005) Molecular physiology of cardiac repolarization. Physiol Rev 85: 1205–1253. 10.1152/physrev.00002.2005 16183911

[3] Bodmer R, Frasch M (2010) Development and Aging of the Drosophila Heart In: Rosenthal N, Harvey R, editors. Heart Development and Regeneration. Amsterdam: Elsevier pp. 47–86.

[4] Vogler G, Bodmer R (2015) Cellular Mechanisms of Drosophila Heart Morphogenesis. J Cardiovasc Dev Dis 2: 2–16. 10.3390/jcdd2010002 26236710 PMC4520698

[5] Ugur B, Chen K, Bellen HJ (2016) Drosophila tools and assays for the study of human diseases. Dis Model Mech 9: 235–244. 10.1242/dmm.023762 26935102 PMC4833332

[6] Ocorr K, Perrin L, Lim HY, Qian L, Wu X, et al (2007) Genetic control of heart function and aging in Drosophila. Trends Cardiovasc Med 17: 177–182. 10.1016/j.tcm.2007.04.001 17574126 PMC1950717

[7] Nishimura M, Ocorr K, Bodmer R, Cartry J (2011) Drosophila as a model to study cardiac aging. Exp Gerontol 46: 326–330. 10.1016/j.exger.2010.11.035 21130861 PMC3079765

[8] Piazza N, Wessells RJ (2011) Drosophila models of cardiac disease. Prog Mol Biol Transl Sci 100: 155–210. 10.1016/B978-0-12-384878-9.00005-4 21377627 PMC3551295

[9] Miller A (1950) The internal anatomy and histology of the imago of Drosophila melanogaster In: Demerc M, editor. Biology of Drosophila. New York: Wiley pp. 420–534.

[10] Bodmer R (1995) Heart development in Drosophila and its relationship to vertebrate systems. Trends in Cardiovascular Medicine: 21–27. 10.1016/1050-1738(94)00032-Q 21232234

[11] Gu GG, Singh S (1995) Pharmacological analysis of heartbeat in Drosophila. J Neurobiol 28: 269–280. 10.1002/neu.480280302 8568510

[12] Dowse H, Ringo J, Power J, Johnson E, Kinney K, et al (1995) A congenital heart defect in Drosophila caused by an action-potential mutation. J Neurogenet 10: 153–168. 8719771 10.3109/01677069509083461

[13] Johnson E, Ringo J, Dowse H (2000) Native and heterologous neuropeptides are cardioactive in Drosophila melanogaster. J Insect Physiol 46: 1229–1236. 10818250 10.1016/s0022-1910(00)00043-3

[14] Johnson E, Sherry T, Ringo J, Dowse H (2002) Modulation of the cardiac pacemaker of Drosophila: cellular mechanisms. J Comp Physiol B 172: 227–236. 10.1007/s00360-001-0246-8 11919704

[15] Wessells RJ, Bodmer R (2007) Age-related cardiac deterioration: insights from Drosophila. Front Biosci 12: 39–48. 17127282 10.2741/2047

[16] Ocorr K, Reeves NL, Wessells RJ, Fink M, Chen HS, et al (2007) KCNQ potassium channel mutations cause cardiac arrhythmias in Drosophila that mimic the effects of aging. Proc Natl Acad Sci U S A 104: 3943–3948. 10.1073/pnas.0609278104 17360457 PMC1820688

[17] Cammarato A, Dambacher CM, Knowles AF, Kronert WA, Bodmer R, et al (2008) Myosin transducer mutations differentially affect motor function, myofibril structure, and the performance of skeletal and cardiac muscles. Mol Biol Cell 19: 553–562 10.1091/mbc.E07-09-0890 18045988 PMC2230588

[18] Cammarato A, Ahrens CH, Alayari NN, Qeli E, Rucker J, et al (2011) A mighty small heart: the cardiac proteome of adult Drosophila melanogaster. PLoS One 6: e18497 10.1371/journal.pone.0018497 21541028 PMC3081823

[19] Wolf MJ, Amrein H, Izatt JA, Choma MA, Reedy MC, et al (2006) Drosophila as a model for the identification of genes causing adult human heart disease. Proc Natl Acad Sci U S A 103: 1394–1399. 10.1073/pnas.0507359103 16432241 PMC1360529

[20] Allikian MJ, Bhabha G, Dospoy P, Heydemann A, Ryder P, et al (2007) Reduced life span with heart and muscle dysfunction in Drosophila sarcoglycan mutants. Hum Mol Genet 16: 2933–2943. 10.1093/hmg/ddm254 17855453

[21] Taghli-Lamallem O, Akasaka T, Hogg G, Nudel U, Yaffe D, et al (2008) Dystrophin deficiency in Drosophila reduces lifespan and causes a dilated cardiomyopathy phenotype. Aging Cell 7: 237–249 10.1111/j.1474-9726.2008.00367.x 18221418 PMC2840698

[22] Neely GG, Kuba K, Cammarato A, Isobe K, Amann S, et al (2010) A global in vivo Drosophila RNAi screen identifies NOT3 as a conserved regulator of heart function. Cell 141: 142–153 10.1016/j.cell.2010.02.023 20371351 PMC2855221

[23] Sanyal S, Jennings T, Dowse H, Ramaswami M (2006) Conditional mutations in SERCA, the Sarco-endoplasmic reticulum Ca2+-ATPase, alter heart rate and rhythmicity in Drosophila. J Comp Physiol B 176: 253–263. 10.1007/s00360-005-0046-7 16320060

[24] Ocorr K, Akasaka T, Bodmer R, Ocorr K, Osgood M (2007) Age-related cardiac disease model of Drosophila. Mech Ageing Dev 128: 112–116. 10.1016/j.mad.2006.11.023 17125816 PMC1850850

[25] Akasaka T, Klinedinst S, Ocorr K, Bustamante EL, Kim SK, et al (2006) The ATP-sensitive potassium (KATP) channel-encoded dSUR gene is required for Drosophila heart function and is regulated by tinman. Proc Natl Acad Sci U S A 103: 11999–12004. 10.1073/pnas.0603098103 16882722 PMC1567687

[26] Nakano Y, Shimizu W (2016) Genetics of long-QT syndrome. J Hum Genet 61: 51–55. 10.1038/jhg.2015.74 26108145

[27] Maljevic S, Wuttke TV, Seebohm G, Lerche H (2010) KV7 channelopathies. Pflugers Arch 460: 277–288. 10.1007/s00424-010-0831-3 20401729

[28] Sanguinetti MC (1999) Dysfunction of delayed rectifier potassium channels in an inherited cardiac arrhythmia. Ann N Y Acad Sci 868: 406–413. 10414310 10.1111/j.1749-6632.1999.tb11302.x

[29] Johnson E, Ringo J, Bray N, Dowse H (1998) Genetic and pharmacological identification of ion channels central to the Drosophila cardiac pacemaker. J Neurogenet 12: 1–24. 9666898 10.3109/01677069809108552

[30] Schmitt N, Grunnet M, Olesen SP (2014) Cardiac potassium channel subtypes: new roles in repolarization and arrhythmia. Physiol Rev 94: 609–653. 10.1152/physrev.00022.2013 24692356

[31] Fink M, Callol-Massot C, Chu A, Ruiz-Lozano P, Izpisua Belmonte JC, et al (2009) A new method for detection and quantification of heartbeat parameters in Drosophila, zebrafish, and embryonic mouse hearts. Biotechniques 46: 101–113. 10.2144/000113078 19317655 PMC2855226

[32] Cammarato A, Ocorr S, Ocorr K (2015) Enhanced assessment of contractile dynamics in Drosophila hearts. Biotechniques 58: 77–80. 10.2144/000114255 25652030 PMC12947757

[33] Straight AF, Cheung A, Limouze J, Chen I, Westwood NJ, et al (2003) Dissecting temporal and spatial control of cytokinesis with a myosin II Inhibitor. Science 299: 1743–1747. 10.1126/science.1081412 12637748

[34] Jou CJ, Spitzer KW, Tristani-Firouzi M (2010) Blebbistatin effectively uncouples the excitation-contraction process in zebrafish embryonic heart. Cell Physiol Biochem 25: 419–424. 10.1159/000303046 20332622 PMC3025892

[35] Dulcis D, Levine RB (2005) Glutamatergic innervation of the heart initiates retrograde contractions in adult Drosophila melanogaster. J Neurosci 25: 271–280. 10.1523/JNEUROSCI.2906-04.2005 15647470 PMC6725498

[36] Lalevee N, Monier B, Senatore S, Perrin L, Semeriva M (2006) Control of cardiac rhythm by ORK1, a Drosophila two-pore domain potassium channel. Curr Biol 16: 1502–1508. 10.1016/j.cub.2006.05.064 16890525

[37] Qu Z, Xie LH, Olcese R, Karagueuzian HS, Chen PS, et al (2013) Early afterdepolarizations in cardiac myocytes: beyond reduced repolarization reserve. Cardiovasc Res 99: 6–15. 10.1093/cvr/cvt104 23619423 PMC3687754

[38] Sanguinetti MC, Jurkiewicz NK (1990) Two components of cardiac delayed rectifier K+ current. Differential sensitivity to block by class III antiarrhythmic agents. J Gen Physiol 96: 195–215. 2170562 10.1085/jgp.96.1.195 PMC2228985

[39] Jurkiewicz NK, Sanguinetti MC (1993) Rate-dependent prolongation of cardiac action potentials by a methanesulfonanilide class III antiarrhythmic agent. Specific block of rapidly activating delayed rectifier K+ current by dofetilide. Circ Res 72: 75–83. 8417848 10.1161/01.res.72.1.75

[40] Alayari NN, Vogler G, Taghli-Lamallem O, Ocorr K, Bodmer R, et al (2009) Fluorescent labeling of Drosophila heart structures. J Vis Exp. 10.3791/1423 PMC3164059 19826399

[41] Kramps T, Peter O, Brunner E, Nellen D, Froesch B, et al (2002) Wnt/wingless signaling requires BCL9/legless-mediated recruitment of pygopus to the nuclear beta-catenin-TCF complex. Cell 109: 47–60. 11955446 10.1016/s0092-8674(02)00679-7

[42] Archbold HC, Yang YX, Chen L, Cadigan KM (2012) How do they do Wnt they do?: regulation of transcription by the Wnt/beta-catenin pathway. Acta Physiol (Oxf) 204: 74–109. 21624092 10.1111/j.1748-1716.2011.02293.x

[43] Cadigan KM (2008) Wnt-beta-catenin signaling. Curr Biol 18: R943–947. 10.1016/j.cub.2008.08.017 18957245

[44] Thompson B, Townsley F, Rosin-Arbesfeld R, Musisi H, Bienz M (2002) A new nuclear component of the Wnt signalling pathway. Nat Cell Biol 4: 367–373. 10.1038/ncb786 11988739

[45] Tang M, Yuan W, Fan X, Liu M, Bodmer R, et al (2013) Pygopus maintains heart function in aging Drosophila independently of canonical Wnt signaling. Circ Cardiovasc Genet 6: 472–480. 10.1161/CIRCGENETICS.113.000253 24046329 PMC3871875

[46] Tang M, Yuan W, Bodmer R, Wu X, Ocorr K (2014) The role of pygopus in the differentiation of intracardiac valves in Drosophila. Genesis 52: 19–28. 10.1002/dvg.22724 24265259 PMC4142678

[47] Rhodes TE, Abraham RL, Welch RC, Vanoye CG, Crotti L, et al (2008) Cardiac potassium channel dysfunction in sudden infant death syndrome. J Mol Cell Cardiol 44: 571–581. 10.1016/j.yjmcc.2007.11.015 18222468 PMC2386856

[48] Witchel HJ (2007) The hERG potassium channel as a therapeutic target. Expert Opin Ther Targets 11: 321–336. 10.1517/14728222.11.3.321 17298291

[49] Sanguinetti MC, Tristani-Firouzi M (2006) hERG potassium channels and cardiac arrhythmia. Nature 440: 463–469. 10.1038/nature04710 16554806

[50] Teng GQ, Zhao X, Lees-Miller JP, Quinn FR, Li P, et al (2008) Homozygous missense N629D hERG (KCNH2) potassium channel mutation causes developmental defects in the right ventricle and its outflow tract and embryonic lethality. Circ Res 103: 1483–1491. 10.1161/CIRCRESAHA.108.177055 18948620 PMC2774899

[51] Papaefthimiou I, Hamilton A, Denti M, Baulcombe D, Tsagris M, et al (2001) Replicating potato spindle tuber viroid RNA is accompanied by short RNA fragments that are characteristic of post-transcriptional gene silencing. Nucleic Acids Res 29: 2395–2400. 11376158 10.1093/nar/29.11.2395 PMC55696

[52] Andrade J, Khairy P, Dobrev D, Nattel S (2014) The clinical profile and pathophysiology of atrial fibrillation: relationships among clinical features, epidemiology, and mechanisms. Circ Res 114: 1453–1468. 10.1161/CIRCRESAHA.114.303211 24763464

[53] Go AS, Mozaffarian D, Roger VL, Benjamin EJ, Berry JD, et al (2013) Executive summary: heart disease and stroke statistics—2013 update: a report from the American Heart Association. Circulation 127: 143–152. 10.1161/CIR.0b013e318282ab8f 23283859

[54] Jindal HK, Merchant E, Balschi JA, Zhangand Y, Koren G (2012) Proteomic analyses of transgenic LQT1 and LQT2 rabbit hearts elucidate an increase in expression and activity of energy producing enzymes. J Proteomics 75: 5254–5265. 10.1016/j.jprot.2012.06.034 22796357 PMC4386689

[55] Wu X, Golden K, Bodmer R (1995) Heart development in Drosophila requires the segment polarity gene wingless. Dev Biol 169: 619–628. 10.1006/dbio.1995.1174 7781903

[56] Vincent SD, Buckingham ME (2010) How to make a heart: the origin and regulation of cardiac progenitor cells. Curr Top Dev Biol 90: 1–41. 10.1016/S0070-2153(10)90001-X 20691846

[57] Gessert S, Kuhl M (2010) The multiple phases and faces of wnt signaling during cardiac differentiation and development. Circ Res 107: 186–199. 10.1161/CIRCRESAHA.110.221531 20651295

[58] Cohen ED, Tian Y, Morrisey EE (2008) Wnt signaling: an essential regulator of cardiovascular differentiation, morphogenesis and progenitor self-renewal. Development 135: 789–798. 10.1242/dev.016865 18263841

[59] Daskalopoulos EP, Hermans KC, Janssen BJ, Matthijs Blankesteijn W (2013) Targeting the Wnt/frizzled signaling pathway after myocardial infarction: a new tool in the therapeutic toolbox? Trends Cardiovasc Med 23: 121–127. 10.1016/j.tcm.2012.09.010 23266229

[60] Pahnke A, Conant G, Huyer LD, Zhao Y, Feric N, et al (2016) The role of Wnt regulation in heart development, cardiac repair and disease: A tissue engineering perspective. Biochem Biophys Res Commun 473: 698–703. 10.1016/j.bbrc.2015.11.060 26626076 PMC4854783

[61] Bhambhani C, Chang JL, Akey DL, Cadigan KM (2011) The oligomeric state of CtBP determines its role as a transcriptional co-activator and co-repressor of Wingless targets. EMBO J 30: 2031–2043. 10.1038/emboj.2011.100 21468031 PMC3098475

[62] Chinchilla A, Franco D (2006) Regulatory mechanisms of cardiac development and repair. Cardiovasc Hematol Disord Drug Targets 6: 101–112. 16787195 10.2174/187152906777441849

[63] Xu J, Cui G, Esmailian F, Plunkett M, Marelli D, et al (2004) Atrial extracellular matrix remodeling and the maintenance of atrial fibrillation. Circulation 109: 363–368. 10.1161/01.CIR.0000109495.02213.52 14732752

[64] Corradi D, Callegari S, Maestri R, Benussi S, Alfieri O (2008) Structural remodeling in atrial fibrillation. Nat Clin Pract Cardiovasc Med 5: 782–796. 10.1038/ncpcardio1370 18852714

[65] Toh N, Morita H, Nagase S, Taniguchi M, Miura D, et al (2010) Atrial electrophysiological and structural remodeling in high-risk patients with Brugada syndrome: assessment with electrophysiology and echocardiography. Heart Rhythm 7: 218–224. 10.1016/j.hrthm.2009.10.035 20129298

[66] Jalife J, Kaur K (2014) Atrial remodeling, fibrosis, and atrial fibrillation. Trends Cardiovasc Med. 10.1016/j.tcm.2014.12.015 PMC5658790 25661032

[67] Kostin S, Klein G, Szalay Z, Hein S, Bauer EP, et al (2002) Structural correlate of atrial fibrillation in human patients. Cardiovasc Res 54: 361–379. 12062341 10.1016/s0008-6363(02)00273-0

[68] Burstein B, Qi XY, Yeh YH, Calderone A, Nattel S (2007) Atrial cardiomyocyte tachycardia alters cardiac fibroblast function: a novel consideration in atrial remodeling. Cardiovasc Res 76: 442–452. 10.1016/j.cardiores.2007.07.013 17720149

[69] Hanna N, Cardin S, Leung TK, Nattel S (2004) Differences in atrial versus ventricular remodeling in dogs with ventricular tachypacing-induced congestive heart failure. Cardiovasc Res 63: 236–244. 10.1016/j.cardiores.2004.03.026 15249181

[70] Andreasen L, Nielsen JB, Christophersen IE, Holst AG, Sajadieh A, et al (2013) Genetic modifier of the QTc interval associated with early-onset atrial fibrillation. Can J Cardiol 29: 1234–1240. 10.1016/j.cjca.2013.06.009 24074973

[71] Wu CF, Ganetzky B, Jan LY, Jan YN, Benzer S (1978) A Drosophila mutant with a temperature-sensitive block in nerve conduction. Proc Natl Acad Sci U S A 75: 4047–4051. 211514 10.1073/pnas.75.8.4047 PMC392928

[72] Titus SA, Warmke JW, Ganetzky B (1997) The Drosophila erg K+ channel polypeptide is encoded by the seizure locus. J Neurosci 17: 875–881. 8994042 10.1523/JNEUROSCI.17-03-00875.1997 PMC6573166

[73] Lo PC, Frasch M (2001) A role for the COUP-TF-related gene seven-up in the diversification of cardioblast identities in the dorsal vessel of Drosophila. Mech Dev 104: 49–60. 11404079 10.1016/s0925-4773(01)00361-6

[74] McGuire SE, Le PT, Osborn AJ, Matsumoto K, Davis RL (2003) Spatiotemporal rescue of memory dysfunction in Drosophila. Science 302: 1765–1768. 10.1126/science.1089035 14657498

[75] Roman G, Endo K, Zong L, Davis RL (2001) P[Switch], a system for spatial and temporal control of gene expression in Drosophila melanogaster. Proc Natl Acad Sci U S A 98: 12602–12607. 10.1073/pnas.221303998 11675496 PMC60100

[76] Venken KJ, Carlson JW, Schulze KL, Pan H, He Y, et al (2009) Versatile P[acman] BAC libraries for transgenesis studies in Drosophila melanogaster. Nat Methods 6: 431–434. 10.1038/nmeth.1331 19465919 PMC2784134

[77] Vogler G, Ocorr K (2009) Visualizing the beating heart in Drosophila. J Vis Exp. 10.3791/1425 PMC3150055 19786947

[78] Ocorr K, Fink M, Cammarato A, Bernstein S, Bodmer R (2009) Semi-automated Optical Heartbeat Analysis of small hearts. J Vis Exp. 10.3791/1435 PMC3150057 19759521

[79] Molina MR, Cripps RM (2001) Ostia, the inflow tracts of the Drosophila heart, develop from a genetically distinct subset of cardial cells. Mech Dev 109: 51–59. 11677052 10.1016/s0925-4773(01)00509-3

[80] Melkani GC, Trujillo AS, Ramos R, Bodmer R, Bernstein SI, et al (2013) Huntington's Disease Induced Cardiac Amyloidosis Is Reversed by Modulating Protein Folding and Oxidative Stress Pathways in the Drosophila Heart. PLoS Genet 9: e1004024 10.1371/journal.pgen.1004024 24367279 PMC3868535

[81] Gautier L, Cope L, Bolstad BM, Irizarry RA (2004) affy—analysis of Affymetrix GeneChip data at the probe level. Bioinformatics 20: 307–315. 10.1093/bioinformatics/btg405 14960456

[82] Emig D, Salomonis N, Baumbach J, Lengauer T, Conklin BR, et al (2010) AltAnalyze and DomainGraph: analyzing and visualizing exon expression data. Nucleic Acids Res 38: W755–762. 10.1093/nar/gkq405 20513647 PMC2896198

[83] Zeitouni B, Senatore S, Severac D, Aknin C, Semeriva M, et al (2007) Signalling pathways involved in adult heart formation revealed by gene expression profiling in Drosophila. PLoS Genet 3: 1907–1921. 10.1371/journal.pgen.0030174 17937502 PMC2014791

[84] Zambon AC, Gaj S, Ho I, Hanspers K, Vranizan K, et al (2012) GO-Elite: a flexible solution for pathway and ontology over-representation. Bioinformatics 28: 2209–2210. 10.1093/bioinformatics/bts366 22743224 PMC3413395

