# Supplementary figures and images for: Age-dependent electrical and morphological remodeling of the Drosophila heart caused by hERG/seizure mutations
Source: PLoS Genet. 2017 May 19;13(5):e1006786. doi: 10.1371/journal.pgen.1006786 (PMC5459509; doi:10.1371/journal.pgen.1006786)

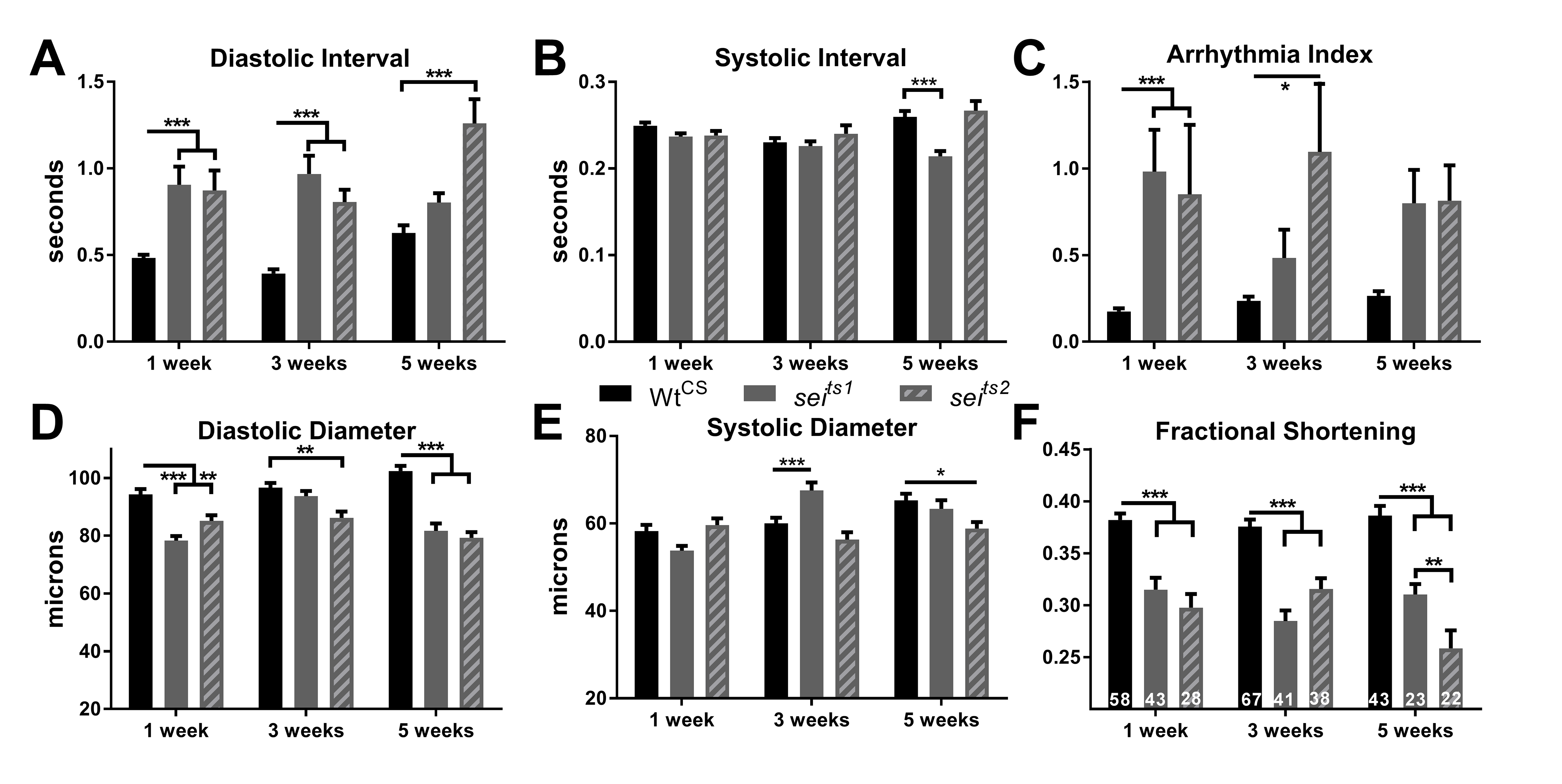

Supplement: S1 Fig — (A) Mean diastolic intervals are significantly increased at all ages in hearts from sei mutants. (B) Mean systolic interval lengths are not significantly affected in sei mutants. (C) Arrhythmia is significantly increased in sei mutants at young ages although the increased arrhythmia at old ages in mutants was not significantly different from WtCS. (D) Hearts from female sei mutants are significantly smaller during diastole that controls at all ages. (E) Systolic diameters did not show consistent differences in hearts from sei mutants. (F) Cardiac contractility, measured as fractional shortening, is significantly reduced at all ages in sei mutants. (TIF) [file pgen.1006786.s001.tif]

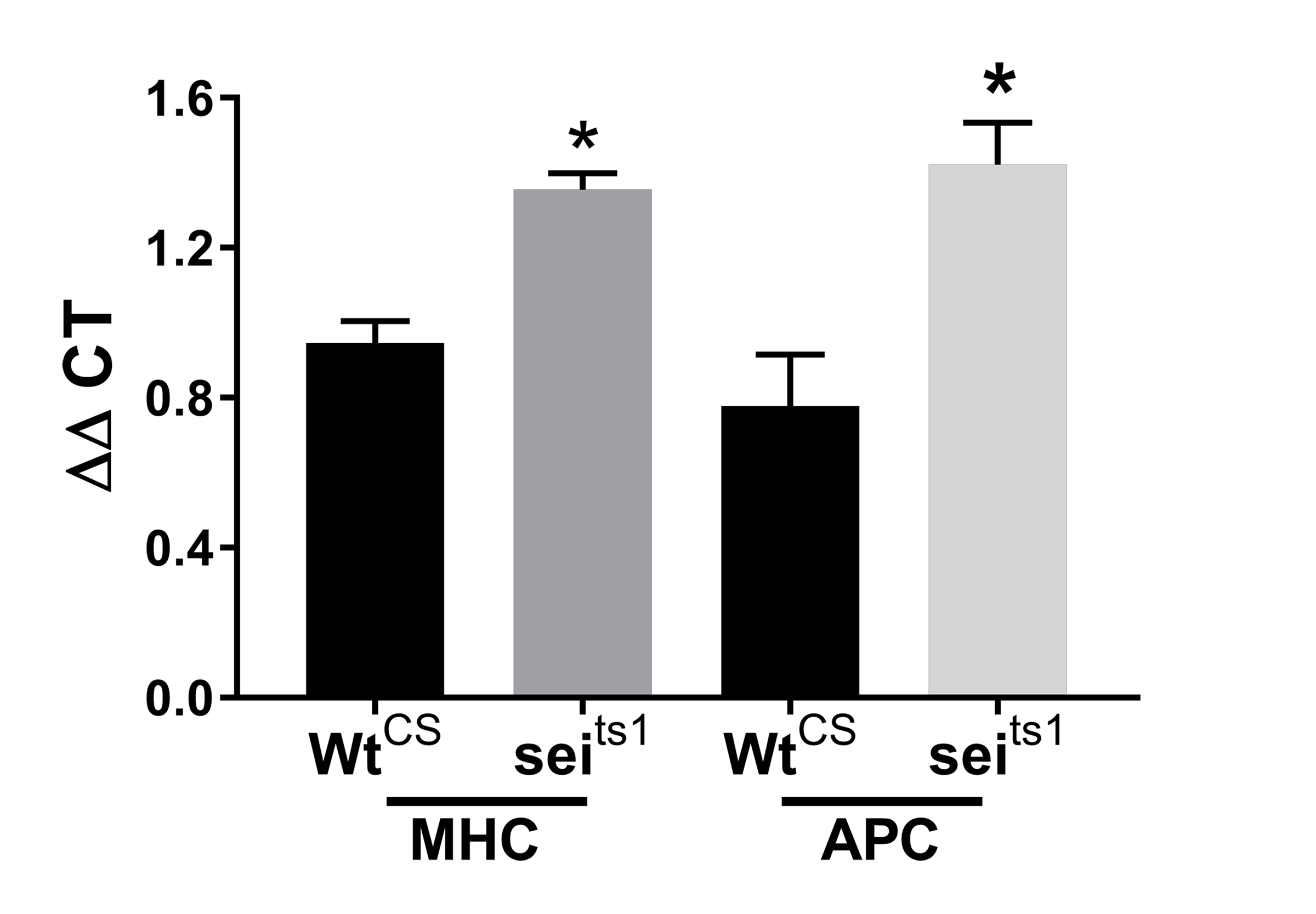

Supplement: S2 Fig — 10–12 pooled isolated hearts from both WtCS and setts1 mutants were subjected to qPCR analysis for actin, myosin heavy chain and APC. ΔΔ CT values were calculated relative to actin for each sample (triplicate experimental replicates and triplicate biological replicates, significance calculated by unpaired t-test, *p,0.05, **p,0.01). Note that Ct values are inversely correlated with relative expression. (TIF) [file pgen.1006786.s002.tif]
