# Supplementary material for: Age-dependent electrical and morphological remodeling of the Drosophila heart caused by hERG/seizure mutations
Source: PLoS Genet. 2017 May 19;13(5):e1006786. doi: 10.1371/journal.pgen.1006786 (PMC5459509; doi:10.1371/journal.pgen.1006786)
Supplement: S1 Table — (DOCX) [file pgen.1006786.s005.docx]

**Supplemental Table 1**

| **PCR Primer Sets:** | | |
| --- | --- | --- |
| **seizure (sei)** | Forward | AATGATAACTCCCGAGACC |
|  | Reverse | CGAGCATAAGTGAGCCCTAAT |
| **slowpoke (slo)** | Forward | GCCAACAGATCAGGTATTCGT |
|  | Reverse | GCGCTACACAGTAACAATCA |
| **shaker (sh)** | Forward | CCGGTCAATGTCCCTTTAGAC |
|  | Reverse | CGGCTTGCGAACTTTC |
| **shaker-like (shal)** | Forward | ATGACGACGCTGGGATATGG |
|  | Reverse | AGGTACAGGTAAGGCGATGAC |
| **inwardly rectifying K+ channel (Ir, IrK1)** | Forward | CGCCGTCTTAGCATCA |
|  | Reverse | CATGGAATTTCGCTTAGCTTA |
| **ether-a-gogo like (elk)** | Forward | CGGGAGTGAGCTACGG |
|  | Reverse | CAGCAGGATGGGTCTTGAGTT |
| **Adenomatous polyposis coli (APC)** | Forward | CAGCAGCCAAGCATTTTCTGG |
|  | Reverse | CAGCAGCCAAGCATTTTCTGG |
| **Actin** | Forward | ATCCGCAAGGATCTGTATGC |
|  | Reverse | ACATCTGCTGGAAGGTGGAC |
| **Myosin Heavy Chain (MHC)** | Forward | ATTTTCGCCATCTCTGACGGT |
|  | Reverse | TTCTTGGTGTTCTCAGTCTTTCC |
| **MHC** | Forward | AAGCCAGTCGCAAATCAGGAG |
|  | Reverse | TTCGAGTCATAGGGTTTCGATTG |
| **Fluidigm Primer Sets:** | | |
| **Ca-alpha1D (CAD)** | Forward | CTACGTCCACTGCGACTTGTA |
|  | Reverse | AGTGGCACCATGGCCTTTAA |
| **Elk** | Forward | CTGCCCTTTGATCACCTGTAC |
|  | Reverse | CAGGAGACGCGTCAATTTCA |
| **Ir** | Forward | GCAACGTTGTGCAGGGAAA |
|  | Reverse | CGTCAACCAGGGTGGTGAA |
| **KCNQ** | Forward | TGAAGCCCTACGACGTCAA |
|  | Reverse | GCATTTTAACGCGACCCAAC |
| **Sei** | Forward | AATCCAGAGAGCCGGCAATA |
|  | Reverse | CCGACCGTTGGGTAAATACAC |
| **Sh** | Forward | CCGAGCTTCGATGCGATTTTA |
|  | Reverse | GGGACATTGACCGGTCTCC |
| **Slo** | Forward | TCATCCAGCTGATGCAGTACC |
|  | Reverse | ATCGTCGCCCTGTTTCCAA |
| **Sulfonylurea Receptor**  **channel (SUR)** | Forward | GCAGCTGAAGGAGTTTGTCA |
|  | Reverse | AGGTTTAGCCCTCCATCACA |
